# ﻿An updated synthesis of the Geophilomorpha (Chilopoda) of Asian Russia

**DOI:** 10.3897/zookeys.1198.119781

**Published:** 2024-04-23

**Authors:** Yurii V. Dyachkov, Lucio Bonato

**Affiliations:** 1 Altai State University, Lenin Avenue, 61, 656049, Barnaul, Russia Altai State University Barnaul Russia; 2 Tomsk State University, Lenin Avenue, 36, 634050, Tomsk, Russia Tomsk State University Tomsk Russia; 3 Western Caspian University, Istiglaliyyat Street, 31, Baku, Azerbaijan Western Caspian University Baku Azerbaijan; 4 Dipartimento di Biologia, Università di Padova, via U. Bassi 58b, 35131 Padova, Italy Università di Padova Padova Italy

**Keywords:** Biodiversity, fauna, Geophilidae, Mecistocephalidae, Russian Far East, Schendylidae, Siberia

## Abstract

A comprehensive overview of the state of knowledge on the ChilopodaGeophilomorpha of the Asian part of Russia is presented, based on the critical revision of all published morphological descriptions and all geographical records. Revised diagnoses for all the 38 nominal species so far reported from Asian Russia are given, with comments on their validity. Among them a total of 18 species are recorded only from this region and many of them from a single locality only. The species belong to Geophilidae s. l., (in the genera *Arctogeophilus*, *Geophilus*, *Pachymerium*, and *Strigamia*), Schendylidae (*Escaryus*), and Mecistocephalidae (*Agnostrup*, *Arrup*, and *Tygarrup*). At least two species have been introduced, namely *Geophilusflavus* and *Tygarrupjavanicus*. The history of studies on the Geophilomorpha in the Asian part of Russia are also summarized.

## ﻿Introduction

The fauna of ChilopodaGeophilomorpha of large part of the Palearctic region, especially the Asian part of Russia, is still badly understood in comparison with other regions. While the species recorded from Europe, including the European part of Russia, have been recently reviewed (Bonato and Minelli 2014; Volkova 2016), available information and records on the Geophilomorpha living in the Asian part of Russia are still scattered in many and relatively old publications. Moreover, the overall knowledge of many species is largely incomplete, with brief descriptions hardly accessible or comparable.

The present paper aims to provide a comprehensive overview of the state of knowledge of Geophilomorpha from Asian Russia, in order to promote and facilitate further taxonomic and faunistic investigations.

## ﻿Material and methods

We searched the entire taxonomic, faunistic, and ecological literature, to the best of our abilities, to retrieve all taxonomic descriptions, morphological data, and occurrence records of Geophilomorpha from the Asian part of Russia.

For the taxonomy and nomenclature, we referred to Bonato et al. (2014) and ChiloBase (Bonato et al. 2016). For each nominal species, we produced a diagnosis by translating and interpreting its original description, other published morphological information reliably referring to the species, all published illustrations, and taxonomic opinions. We also added diagnoses for the genera. However, for the diagnoses of families we refer to Bonato (2011).

**Figure 1.**
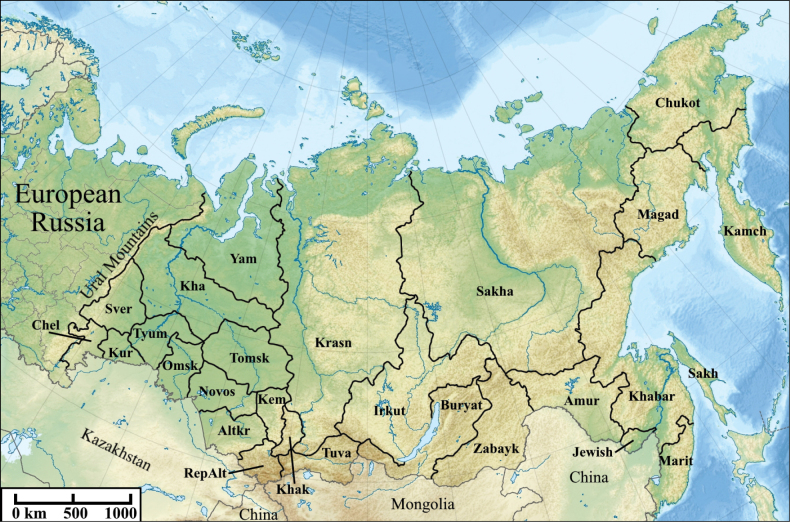
Administrative units of Asian Russia. Western Siberia: Chel – Chelyabinsk oblast, Sver – Sverdlovsk oblast, Kha – Khanty-Mansi autonomous okrug, Yam – Yamalo-Nenets autonomous okrug, Kur – Kurgan oblast, Tyum – Tyumen oblast, Omsk – Omsk oblast, Tomsk – Tomsk oblast, Novos – Novosibirsk oblast, Altkr – Altai krai, RepAlt – Republic of Altai, Kem – Kemerovo oblast, Khak – Republic of Khakassia; Eastern Siberia: Tuva – Republic of Tuva, Krasn – Krasnoyarsk krai, Irkut – Irkutsk oblast, Buryat – Republic of Buryatia, Zabayk – Zabaykalsky krai, Sakha – Republic of Sakha (Yakutia); Far East: Amur – Amur oblast, Khabar – Khabarovsk krai, Magad – Magadan oblast, Chukot – Chukotka autonomous okrug, Kamch – Kamchatka krai, Jewish – Jewish autonomous oblast, Marit – Maritime krai, Sakh – Sakhalin oblast.

We referred the occurrence records to modern administrative units (Fig. 1). The translation of names of Russian administrative divisions into English follows Kazantsev (2022). The Asian part of Russia is a huge region from the Ural Mountains in the west to the Pacific Ocean in the east (Gvozdetskiy and Mikhailov 1978). It spans an area of 13.1 million square kilometers and is often divided into three regions: Western Siberia, Eastern Siberia, and Far East. Western Siberia (approximately between the Urals and the Yenisei River) includes the following administrative units: Yamalo-Nenets and Khanty-Mansi autonomous okrugs; Sverdlovsk, Chelyabinsk, Kurgan, Tyumen, Omsk, Tomsk, Novosibirsk, and Kemerovo oblasts; Altai krai; republics of Altai and Khakassia. Eastern Siberia (approximately between the Yenisei River and the watersheds that run parallel to the coast of the Pacific Ocean) includes the following units: Krasnoyarsk and Zabaykalsky krais; Irkutsk oblast; republics of Tuva, Buryatia, and Sakha (Yakutia); the western parts of Khabarovsk krai, Magadan oblast, and Chukotka autonomous okrug. Russian Far East (river basins flowing into the Pacific Ocean, as well as Wrangel, Commander, Shantar Islands, the Kuril, and Sakhalin) includes the following units: Amur and Sakhalin oblasts, the eastern part of Magadan oblast; Jewish autonomous oblast; the eastern part of Chukotka autonomous okrug; Kamchatka, Maritime krais, and the eastern part of Khabarovsk krai.

Families, genera within families, and species within genera are listed alphabetically (see also Table 1). For each species, we report the type locality/ies, both as indicated in the original publications and with their modern names; the estimated coordinates of the type locality/ies (only for species described from Asian Russia); information on the type material, including the depository (only for species described from Asian Russia or adjacent territories); references to all sources of records for Asian Russia, and to other selected publications that are relevant for the taxonomy, morphology, and distribution of the species; the distribution within Asian Russia and outside; and finally, remarks on the taxonomic status and distribution.

**Table 1.** Nominal species of Geophilomorpha reported from Asian Russia: i – anthropochore introduction, * – uncertain taxonomic validity, ! – known from Asian Russia only.

№	Species	European Russia	Asian Russia		
			Western Siberia	Eastern Siberia	Far East
Family Geophilidae Leach, 1816					
Genus *Arctogeophilus* Attems, 1909					
1	*A.glacialis* (Attems, 1909)				+
2	*A.macrocephalus* Folkmanová & Dobroruka, 1960 *	+	+	+	+
3	*A.sachalinus* Verhoeff, 1934 * !				+
Genus *Geophilus* Leach, 1814					
4	*G.bipartitus* Takakuwa, 1937 !				+
5	*G.flavus* (De Geer, 1778)	+	+i	+	
6	*G.orientalis* Sseliwanoff, 1881 * !				+
7	*G.proximus* C.L. Koch, 1847	+	+		
8	*G.rhomboideus* Takakuwa, 1937				+
9	*G.sibiricus* Stuxberg, 1876 * !			+	
10	*G.sounkyoensis* Takakuwa, 1937				+
Genus *Pachymerium* C.L. Koch, 1847					
11	*P.ferrugineum* (C.L. Koch, 1835)	+	+		+
12	*P.pilosum* (Meinert, 1870) * !			+	+
Genus *Strigamia* Gray, 1843					
13	S.cf.acuminata (Leach, 1815)	+			+
14	*S.alokosternum* (Attems, 1927)				+
15	*S.hirsutipes* (Attems, 1927) *				+
16	*S.pusilla* (Sseliwanoff, 1884)	+	+	+	
17	*S.sacolinensis* (Meinert, 1870) * !				+
18	*S.sibirica* (Sseliwanoff, 1881) * !			+	
19	*S.sulcata* (Sseliwanoff, 1881) * !				+
20	S.cf.transsilvanica (Verhoeff, 1928)		+		+
Family Mecistocephalidae Bollman, 1893					
Genus *Agnostrup* Foddai, Bonato, Pereira & Minelli, 2003					
21	*A.striganovae* (Titova, 1975) !				+
Genus *Arrup* Chamberlin, 1912					
22	*A.dentatus* (Takakuwa, 1934)				+
23	*A.mamaevi* (Titova, 1975) !				+
Genus *Tygarrup* Chamberlin, 1914					
24	*T.javanicus* Attems, 1929		+i		
Family Schendylidae Cook, 1896					
Genus *Escaryus* Cook & Collins, 1891					
25	*E.chadaevae* Titova, 1973	+	+	+	
26	*E.chichibuensis* Shinohara, 1955 *				+
27	*E.dentatus* Titova, 1973 * !				+
28	*E.hirsutus*Titova 1973 * !				+
29	*E.japonicus* Attems, 1927 *	+	+	+	+
30	*E.koreanus* Takakuwa, 1937 *		+	+	+
31	*E.krivolutskiji* Titova, 1973 * !				+
32	*E.molodovae* Titova, 1973 * !				+
33	*E.perelae* Titova, 1973 * !				+
34	*E.polygonatus* Titova, 1973 * !				+
35	*E.retusidens* Attems, 1904	+	+		+
36	*E.sachalinus* Takakuwa, 1935				+
37	*E.sibiricus* Cook, 1899 !				+
38	*E.vitimicus* Titova, 1973 * !			+	

An asterisk (*) indicates nominal species whose taxonomic validity requires confirmation.

### ﻿Abbreviations for depositories

**NHMD** Natural History Museum of Denmark, Copenhagen;

**NHMW**Natural History Museum Vienna (Austria);

**ZISP**Zoological Institute of the Russian Academy of Sciences (Saint Petersburg, Russia);

**ZMH**Zoological Museum in Hamburg (Germany);

**ZMMU**Zoological Museum of the Moscow State University (Moscow, Russia).

## ﻿Results

**Family Geophilidae Leach, 1816**

### ﻿Genus Arctogeophilus

Taxon classificationAnimaliaGeophilomorphaGeophilidae

Attems, 1909

6F2BA905-9224-5C7D-8332-814C96F97956

#### Diagnosis.

Geophilids with head distinctly elongate; clypeal areas present, variously distinct; labral side-pieces almost touching medially; second maxillary coxosternite medially very short and poorly sclerotized, with statuminia, without anterior inner processes; second maxillary pretarsus claw-like; forcipular tergite distinctly narrower than subsequent tergite, with pleurites exposed dorsally; forcipular coxosternite relatively broad posteriorly, without anterior denticles, with chitin-lines short or absent, with coxopleural sutures subparallel in their anterior half; forcipular trochanteroprefemur distinctly elongate, with distal denticle; forcipular tarsungulum with basal denticle; trunk sternites without “carpophagus” structures; ventral pore-fields usually absent; metasternite of ultimate leg-bearing segment longer than wide; coxopleura usually with sparse pores; legs of the ultimate pair longer than the penultimate legs, often without pretarsus. See Table 2.

**Table 2.** Differences between species of the genus *Arctogeophilus* Attems, 1909 known from Asian Russia and adjacent territories.

Species	Characters		
	First maxillary lappets	Denticles on forcipular intermediate articles	Ventral pore-fields
*A.glacialis* (Attems, 1909)	short	slightly shorter than other denticles	absent
*A.macrocephalus* Folkmanová & Dobroruka, 1960	long	smaller than other denticles	on some anterior segments
*A.sachalinus* Verhoeff, 1934	long	absent	on some anterior segments
*A.attemsi* Folkmanová, 1956	long	absent	absent

### ﻿1. Arctogeophilus glacialis

Taxon classificationAnimaliaGeophilomorphaGeophilidae

(Attems, 1909)

9E8F305F-55C8-5514-A9EA-9A6C4A498E17

Geophilus (Arctogeophilus) glacialis Attems 1909: 23.
Arctogeophilus glacialis – Attems 1929: 297; Chamberlin 1946: 182.
Cryophilus alaskanus Chamberlin 1919: 18 (synonymy by Chamberlin 1946: 182).

#### Type localities.

Russia: Chukotka autonomous okrug: “Nunamo” (Attems 1909; see Remarks), “Konyam Bay im Senjavin Sund” (Attems 1909) = Penkigney Bay, ca 64°49'N, 172°53'W; USA: Alaska: “Port Clarence” (Attems 1909), ca 65°15'N, 166°51'W.

#### Type series.

***Syntypes***: 7 specimens, including 3 males and 4 females. Deposited in NHMW (Ilie et al. 2009).

#### Diagnosis.

A species of *Arctogeophilus* with first maxillary lappets relatively short; denticles on all forcipular articles; denticles on the forcipular intermediate articles only slightly shorter than those on trochanteroprefemur and tarsungulum; 39 leg-bearing segments, possibly invariably; ventral pore-fields absent; pretarsus of ultimate legs absent.

#### Distribution.

Far East: Chukotka autonomous okrug (Attems 1909). Outside Asian Russia: Alaska and Canada (e.g., Attems 1909; Chamberlin 1946; Langor and Langor 2022).

#### Remarks.

The position of the locality “Nunamo” (indicated by Attems 1909) is uncertain: the original material was collected during the Vega expedition, and Nordenskiöld (1882: 565, 567) mentioned a tent-village “Nunamo” located in Chukotka, but he did not indicate the precise position of this place. Instead, Nordenskiöld (1882: 565) provided coordinates for “Konyam Bay” = Penkigney Bay.

### ﻿2. Arctogeophilus macrocephalus

Taxon classificationAnimaliaGeophilomorphaGeophilidae

Folkmanová & Dobroruka, 1960 *

9C967986-F32F-54FF-B8C5-125B5FAA5838

Arctogeophilus macrocephalus Folkmanová & Dobroruka, 1960: 1815.
Arctogeophilus sp. – Byzova and Chadaeva 1965: 333.
Arctogeophilus macrocephalus – Zalesskaja et al. 1982: 188; Ganin 1997: 105, 109, 112, 114, 129, 134, 141; Vorobiova 1999: 33; 2002: 63; Rybalov 2002: 83; Farzalieva 2008: 56; Volkova 2016: 671; Nefediev et al. 2017a: 8; 2017c: 221; 2018: 236; 2021: 37; Dyachkov and Tuf 2019: 25; Nefediev 2019: 24.

#### Type locality.

Russia: Republic of Tatarstan: Chistopolsky District: “Bliz s. Zmievo” (Folkmanová and Dobroruka 1960) = near Zmievo village, ca 55°23'N, 50°43'E.

#### Type series.

***Syntypes***: 20 specimens. Depository unknown.

#### Diagnosis.

A species of *Arctogeophilus* with first maxillary lappets relatively long; denticles on all forcipular articles, those on intermediate articles smaller than those on trochanteroprefemur and tarsungulum; 35–43 leg-bearing segments; ventral pore-fields on some anterior segments; pretarsus of ultimate legs absent.

#### Distribution.

Western Siberia: Sverdlovsk, Chelyabinsk, Tomsk, and Kemerovo oblasts, Altai krai, republics of Altai and Khakassia (Byzova and Chadaeva 1965; Zalesskaja et al. 1982; Farzalieva 2008; Nefediev et al. 2017a, 2017c, 2018, 2021; Nefediev 2019). Eastern Siberia: Republic of Tuva and Krasnoyarsk krai (Zalesskaja et al. 1982; Vorobiova 1999; Rybalov 2002; Vorobiova et al. 2002). Far East: Jewish autonomous oblast, Amur oblast, Khabarovsk and Maritime krais, Chukotka autonomous okrug, and Sakhalin oblast (Sakhalin and Kunashir islands) (Zalesskaja et al. 1982; Ganin 1997). Outside Asian Russia: westwards to Transcarpathia (Zalesskaja et al. 1982; Volkova 2016) and Eastern Kazakhstan (Dyachkov and Tuf 2019).

#### Remarks.

Zalesskaja et al. (1982: 189) suggested this species can be a junior synonym of *A.glacialis* (Attems, 1909), and this is still in doubt (Nefediev et al. 2017a). Because of this uncertainty, the actual taxonomic status of the populations of *Arctogeophilus* from the Far East remains to clarify.

The type locality for *A.macrocephalus* has been sometimes reported erroneously in previous publications (e.g., Bonato and Minelli 2014).

### ﻿3. Arctogeophilus sachalinus

Taxon classificationAnimaliaGeophilomorphaGeophilidae

Verhoeff, 1934 *

BBDBEAA2-73ED-5DF0-8530-C526A8FBE59D

Arctogeophilus sachalinus Verhoeff 1934: 15.
Arctogeophilus sachalinus – Takakuwa 1940: 135; Molodova 1973: 67; Kurcheva 1977: 45; Zalesskaja et al. 1982: 188; Ganin 1997: 121, 124, 126, 128, 134.

#### Type locality.

Russia: Sakhalin oblast: “Insel Sachalin” (Verhoeff 1934) = Sakhalin Isl.

#### Type series.

***Holotype***: male. Depository unknown.

#### Diagnosis.

A species of *Arctogeophilus* with first maxillary lappets relatively long; forcipular denticles only on the trochanteroprefemur and tarsungulum, not on the intermediate articles; 39 leg-bearing segments, possibly invariably; ventral pore-fields on some anterior segments; pretarsus of ultimate legs absent.

#### Distribution.

Far East: Sakhalin oblast (Sakhalin Isl.) (Verhoeff 1934; Takakuwa 1940; Molodova 1973; Kurcheva 1977; Ganin 1997), Maritime krai (Kurcheva 1977; Ganin 1997), and Chukotka autonomous okrug (Kurcheva 1977). Outside Asian Russia: no records.

#### Remarks.

It has been suggested that *A.sachalinus* could be a junior synonym of *A.glacialis* (Attems, 1909) (Nefediev et al. 2017a). More generally, the actual taxonomic status of the populations of *Arctogeophilus* from the Far East remains to clarify.

### ﻿Genus Geophilus

Taxon classificationAnimaliaGeophilomorphaGeophilidae

Leach, 1814

929DCE0C-5B28-570F-8605-78BD3EEA1135

#### Diagnosis.

Geophilids with head usually only slightly elongate; clypeal areas usually not distinct; labral side-pieces distinctly separated by an intermediate part; second maxillary coxosternite medially long and sclerotized, without both statuminia and anterior inner processes; second maxillary pretarsus claw-like or reduced; forcipular tergite approximately as broad as the subsequent tergite, covering most part of the pleurites; forcipular coxosternite usually wider than long, gradually narrowing posteriorly, without anterior denticles, with chitin-lines, with coxopleural sutures diverging anteriorly also in their anterior half; forcipular trochanteroprefemur only moderately elongate, usually without denticles; forcipular tarsungulum with at most a small basal denticle; trunk sternites often with “carpophagus” pits and often with ventral pore-fields, usually a transverse band on the posterior part of the sternite; metasternite of the ultimate leg-bearing segment usually wider than long; coxopleura with sparse ventral pores, most of them close to metasternite; pretarsus of ultimate leg pair claw-like or reduced. See Table 3.

**Table 3.** Differences between species of the genus *Geophilus* Leach, 1814 known from Asian Russia.

Species	Characters				
	Leg-bearing segments	“Carpophagus” pits	Ventral pore-fields on the anterior metasternites	Coxal pores	Anal pores
*G.bipartitus* Takakuwa, 1937	35–39	present	transverse diamond	all close to the margin of metasternite	present
*G.flavus* (De Geer, 1778)	37–61	absent	transverse band	all close to the margin of metasternite	present
*G.orientalis* Sseliwanoff, 1881	39	?	?	on the ventral and lateral sides of coxopleura	present
*G.proximus* C.L. Koch, 1847	45–55	present	an entire posterior diamond	all close to the margin of metasternite	present
*G.rhomboideus* Takakuwa, 1937	43–49	present	transverse diamond	most pores close to the margin of metasternite and one pore located separately	present
*G.sibiricus* Stuxberg, 1876	57–59	?	?	on the ventral and lateral sides of coxopleura	absent
*G.sounkyoensis* Takakuwa, 1937	55–57	present	transverse band and sparse pores	most pores close to the margin of metasternite and one pore located separately	present

### ﻿4. Geophilus bipartitus

Taxon classificationAnimaliaGeophilomorphaGeophilidae

Takakuwa, 1937

E0FDB8A1-9C40-5194-B76F-8730298755D0

Geophilus bipartitus Takakuwa 1937b: 285.
Geophilus bipartitus – Takakuwa 1937c: 80; 1940: 104; Molodova 1973: 67; Kurcheva 1977: 45.

#### Type locality.

Russia: Sakhalin oblast: Sakhalin Isl.: “Otako (Chikuka)” (see Remarks) (Takakuwa 1937c).

#### Type series.

***Syntypes***: unknown number of specimens, both sexes. Depository unknown.

#### Diagnosis.

A species of *Geophilus* with head slightly longer than wide, 35–39 leg-bearing segments; “carpophagus” pits present, up to as wide as the metasternites; ventral pore-fields present, an entire posterior diamond on the anterior metasternites, absent on most of the posterior metasternites, two paired posterior groups on the penultimate metasternite; metasternite of ultimate leg-bearing segment wider than long; a few coxal pores on each coxopleuron, all close to the margin of metasternite; pretarsus of ultimate leg pair claw-like; anal pores present.

#### Distribution.

Far East: Sakhalin oblast (Sakhalin and Kurile Islands) (Takakuwa 1937b; Molodova 1973; Kurcheva 1977). Outside Asian Russia: no records.

#### Remarks.

The current identity and position of the locality “Otako (Chikuka)” (indicated by Takakuwa 1937c) is uncertain.

### ﻿5. Geophilus flavus

Taxon classificationAnimaliaGeophilomorphaGeophilidae

(De Geer, 1778)

A7EFD629-4BCE-5117-AB30-583CDD5D2894

Geophilus longicornis Leach 1815: 386 (synonymy by Stuxberg 1871: 508).
Arthronomalus longicornis – Gerstfeldt 1859: 275; Sseliwanoff 1884: 90.
Necrophloeophagus longicornis – Brolemann 1930: 151.
Schizotaenia ornata Folkmanová & Dobroruka, 1960: 1816 (synonymy by Bonato and Minelli 2014: 41).
Geophilus flavus – Nefediev et al. 2017a: 9.

#### Type locality.

Unknown, possibly in France (Bonato and Minelli 2014).

#### Diagnosis.

A species of *Geophilus* with head ~ 1.2× as long as wide; antennal articles ≤ 2× as long as wide; second maxillary pretarsus claw-like, longer than surrounding setae; forcipular trochanteroprefemur ~ 1.5× as long as wide; forcipular trochanteroprefemur, femur, and tibia without denticles; tarsungulum bearing a small basal denticle; 37–61 leg-bearing segments; “carpophagus” pits absent; ventral pore-fields present, an entire transverse posterior band on the anterior metasternites, two paired posterior groups on the posterior metasternites; metasternite of ultimate leg-bearing segment wider than long; up to a dozen coxal pores on each coxopleuron, all close to the margin of metasternite; pretarsus of ultimate leg pair claw-like; anal pores present.

#### Distribution.

Western Siberia: Tomsk oblast (Nefediev et al. 2017a). Eastern Siberia: Zabaykalsky krai (Gerstfeldt 1859, as *Arthronomaluslongicornis*). Outside Asian Russia: Western Palaearctic (e.g., Bonato et al. 2005; Stoev 2007).

#### Remarks.

The record from Western Siberia seems to be due to anthropochore introduction (Nefediev et al. 2017a), while the record from Eastern Siberia requires confirmation (see Discussion).

### ﻿6. Geophilus orientalis

Taxon classificationAnimaliaGeophilomorphaGeophilidae

Sseliwanoff, 1881 *

8032F012-AA05-58A3-8D7E-83F56A71DFAD

Geophilus orientalis Sseliwanoff 1881: 4.
Geophilus orientalis – Sseliwanoff 1884: 80; Attems 1903a: 45; 1903b: 235; 1929: 329.

#### Type locality.

Russia: Khabarovsk krai: “Nikolaevsk na Amure” (Sseliwanoff 1881) = Nikolayevsk-on-Amur city, 53°8'N, 140°42'E.

#### Type series.

***Holotype***: male. Deposited in ZISP.

#### Diagnosis.

A species of *Geophilus* with head slightly longer than wide; forcipular coxosternite with short and wide denticles; all forcipular articles with small denticles, except the tarsungulum; 39 leg-bearing segments; metasternite of ultimate leg-bearing segment longer than wide, only slightly narrowing backwards; numerous coxal pores, on the ventral and lateral sides of coxopleura; anal pores present.

#### Distribution.

Far East: Khabarovsk krai (Sseliwanoff 1881, 1884). Outside Asian Russia: no records.

#### Remarks.

The species was originally described under the genus *Geophilus*, but its taxonomic position is considered uncertain since Attems (1929). The few morphological characters reported in the descriptions and keys by Sseliwanoff (1881, 1884) do not allow it to be assigned confidently to one of the known genera.

### ﻿7. Geophilus proximus

Taxon classificationAnimaliaGeophilomorphaGeophilidae

C.L. Koch, 1847

E87A87DC-F076-56AE-B3D1-88344244FD4B

Geophilus proximus – Sseliwanoff 1881: 6. 1884: 87; Titova 1969: 165; Zalesskaja et al. 1982: 188; Poryadina 1991: 14; Striganova and Poryadina 2005: 130; Farzalieva 2008: 58; Bukhkalo and Sergeeva 2012: 61; Bukhkalo et al. 2014: 73; Sergeeva 2013: 530; 2014: 72; Volkova 2016: 673; Nefediev et al. 2017a: 9; 2017b: 114; Dyachkov and Tuf 2019: 25; Nefediev 2019: 24; Bragina et al. 2020: 30.

#### Type locality.

Germany: near Regensburg (Bonato and Minelli 2014).

#### Diagnosis.

A species of *Geophilus* with head slightly longer than wide; antennal articles ≤ ~ 1.5× as long as wide; second maxillary pretarsus claw-like, longer than surrounding setae; forcipular trochanteroprefemur slightly longer than wide; forcipular trochanteroprefemur, femur, and tibia without denticles; forcipular tarsungulum bearing a small basal denticle; 45–55 leg-bearing segments; “carpophagus” pits present, up to as wide as the metasternites; ventral pore-fields present, an entire posterior diamond on the anterior metasternites, absent on the posterior metasternites; metasternite of ultimate leg-bearing segment wider than long; up to a dozen coxal pores on each coxopleuron, all close to the margin of metasternite; pretarsus of ultimate leg pair claw-like; anal pores present.

#### Distribution.

Western Siberia: “Western Siberia” (Sseliwanoff 1881); Sverdlovsk, Chelyabinsk, Tyumen, Omsk, Novosibirsk, Kemerovo, and Tomsk oblasts, Altai krai, Republic of Khakassia (Zalesskaja et al. 1982; Poryadina 1991; Striganova and Poryadina 2005; Farzalieva 2008; Bukhkalo and Sergeeva 2012; Sergeeva 2013, 2014; Bukhkalo et al. 2014; Nefediev et al. 2017a, 2017b, 2021; Nefediev 2019). Outside Asian Russia: Northern Europe (Bonato et al. 2005).

### ﻿8. Geophilus rhomboideus

Taxon classificationAnimaliaGeophilomorphaGeophilidae

Takakuwa, 1937

631E431A-3C27-59E1-BF7E-870354A8AE56

Geophilus rhomboideus Takakuwa 1937b: 284.
Geophilus rhomboideus – Takakuwa 1937c: 78; 1940: 101; Kurcheva 1977: 45.

#### Type localities.

Russia: Sakhalin oblast: “Sachalin (Tomaruoru (= Tomari сity, 47°45'N, 142°3'E), Maoka (= Kholmsk city, 47°02'N, 142°02'E), Sirutori (= Makarov сity, 48°37'N, 142°46'E))” (Takakuwa 1937c).

#### Type series.

***Syntypes***: unknown number of specimens, both sexes. Depository unknown.

#### Diagnosis.

A species of *Geophilus* with head approximately as long as wide; second maxillary pretarsus claw-like and relatively long; forcipular trochanteroprefemur, femur, and tibia without denticles; forcipular tarsungulum bearing a small basal denticle; 43–49 leg-bearing segments; “carpophagus” pits present, up to as wide as the metasternites; ventral pore-fields present, an entire posterior diamond on the anterior metasternites; metasternite of ultimate leg-bearing segment wider than long; a few coxal pores on each coxopleuron, most of them close to the margin of metasternite and one pore located separately; pretarsus of ultimate leg pair claw-like; anal pores present.

#### Distribution.

Far East: Sakhalin oblast (Sakhalin Isl.) (Takakuwa 1937b, 1937c; Kurcheva 1977). Outside Asian Russia: Japan (Takakuwa 1937c).

### ﻿9. Geophilus sibiricus

Taxon classificationAnimaliaGeophilomorphaGeophilidae

Stuxberg, 1876 *

CA206DCA-E862-5A2B-9621-EA929AE50C20

Geophilus sibiricus Stuxberg 1876a: 31.
Geophilus sibiricus – Stuxberg 1876b: 315; Sseliwanoff 1884: 90; Attems 1903a: 45; 1903b: 235; 1929: 329.

#### Type locality.

Russia: Krasnoyarsk krai: “Krasnojarsk” (Stuxberg 1876b) = Krasnoyarsk city, 56°0'N, 92°53'E.

#### Type series.

***Syntypes***: 3 females. Depository unknown.

#### Diagnosis.

A species of *Geophilus* with head ~ 1.2× as long as wide; tarsungulum without basal denticle; 57–59 leg-bearing segments; more than a dozen coxal pores on each coxopleuron, both on the ventral and lateral sides; pretarsus of ultimate leg pair claw-like; anal pores absent.

#### Distribution.

Eastern Siberia: Krasnoyarsk krai (Stuxberg 1876b). Outside Asian Russia: no records.

#### Remarks.

The species was originally described under the genus *Geophilus*, but its taxonomic position was considered uncertain since Attems (1929). The few morphological characters reported in the descriptions by Stuxberg (1876a, 1876b) do not allow to confidently assign it to one of the known genera.

### ﻿10. Geophilus sounkyoensis

Taxon classificationAnimaliaGeophilomorphaGeophilidae

Takakuwa, 1937

78F6346A-F40F-5268-BD98-D6F2104E2EB2

Geophilus sounkyoensis Takakuwa 1937b: 283.
Geophilus sounkyoensis – Takakuwa 1937c: 77; 1940: 99; Ghilarov and Perel 1973: 46; Kurcheva 1977: 45; Ganin 1997: 121, 124, 126, 128.

#### Type locality.

Japan: Hokkaido: “Sounkyo” (Takakuwa 1937c).

#### Type series.

***Syntypes***: unknown number of specimens, both sexes. Depository unknown.

#### Diagnosis.

A species of *Geophilus* with second maxillary pretarsus claw-like, longer than surrounding setae; forcipular trochanteroprefemur slightly longer than wide; forcipular trochanteroprefemur, femur and tibia without denticles; forcipular tarsungulum bearing a small basal denticle; 55–57 leg-bearing segments; “carpophagus” pits present, up to as wide as the metasternites; ventral pore-fields present, an entire posterior band and other sparse pores on the anterior metasternites, absent on the posterior metasternites; metasternite of ultimate leg-bearing segment wider than long; a few coxal pores on each coxopleuron, most of them close to the margin of metasternite and one pore located separately; anal pores present.

#### Distribution.

Far East: Maritime krai (Ghilarov and Perel 1973; Kurcheva 1977; Ganin 1997). Outside Asian Russia: Japan (Takakuwa 1937b, 1937c, 1940).

### ﻿Genus Pachymerium

Taxon classificationAnimaliaGeophilomorphaGeophilidae

C.L. Koch, 1847

18CD5CEC-4B6D-5CEF-A456-7FB049A3AC77

#### Diagnosis.

Geophilids with head distinctly elongate; clypeal areas distinct; labral side-pieces distinctly separated by an intermediate part; second maxillary coxosternite medially long and sclerotized, without both statuminia and anterior inner processes; second maxillary pretarsus claw-like; forcipular tergite distinctly narrower than subsequent tergite, with pleurites exposed dorsally; forcipular coxosternite relatively broad posteriorly, with coxopleural sutures subparallel in their anterior half; forcipular trochanteroprefemur distinctly elongate, with distal denticle; chitin-lines present but short; forcipular tarsungulum with basal denticle; trunk sternites without “carpophagus” structures; ventral pore-fields present at least on the anterior part of the trunk, two paired anterior groups and a posterior entire transverse band on each sternite; metasternite of ultimate leg-bearing segment approximately as long as wide or longer than wide; coxopleura with sparse pores; legs of the ultimate pair with claw-like pretarsus. See Table 4.

**Table 4.** Differences between species of the genus *Pachymerium* C.L. Koch, 1847 known from Asian Russia.

Species	Characters			
	Chitin-lines	Denticle on forcipular tarsungulum	Leg-bearing segments	Ultimate metasternite: lateral margins
*P.ferrugineum* (C.L. Koch, 1835)	extending for most part of the length of the coxosternite	yes	41–69	distinctly converging posteriorly
*P.pilosum* (Meinert, 1870)	very short	no	45–49	subparallel

### ﻿11. Pachymerium ferrugineum

Taxon classificationAnimaliaGeophilomorphaGeophilidae

(C.L. Koch, 1835)

425E6327-7C3B-54CD-B8A4-1690CAF597D1

Geophilus ferrugineus – Sseliwanoff 1884: 77.
Pachymerium ferrugineum – Titova 1969: 166; Zalesskaja et al. 1982: 187; Ganin 1997: 105, 112, 116, 146; Farzalieva 2008: 61; Sergeeva 2013: 530; Zuev 2016: 36; Zuev and Evsyukov 2016: 424; Bukhkalo et al. 2014: 73; Volkova 2016: 673; Dyachkov 2018a: 252; Nefediev et al. 2017a: 11; Dyachkov and Tuf 2019: 26; Dyachkov 2020: 79; 2022: 71; 2023: 1077; Dyachkov and Nedoev 2021: 42; Dyachkov et al. 2022: 73; 2023: 63; Dyachkov and Farzalieva 2023: 228.

#### Type locality.

Germany: Arklee, near Regensburg (Bonato and Minelli 2014).

#### Diagnosis.

A species of *Pachymerium* with two paired clypeal areas; forcipular coxosternite with chitin-lines, which extend for most part of the length of the coxosternite, but do not reach the anterior margin; both forcipular trochanteroprefemur and tarsungulum with relatively small denticles; 41–69 leg-bearing segments; ventral pore-fields present, also on the posterior part of the trunk, where they are reduced to two paired posterior groups on each metasternite; ultimate metasternite trapezoidal, approximately as long as wide, distinctly tapering towards the posterior margin; all coxal pores sparse from the ventral to the dorsal sides of the coxopleura.

#### Distribution.

Western Siberia: Chelyabinsk, Sverdlovsk, Tyumen, and Tomsk oblasts, Altai krai (Zalesskaja et al. 1982; Farzalieva 2008; Sergeeva 2013; Bukhkalo et al. 2014; Nefediev et al. 2017a). Far East: Amur oblast, Jewish autonomous oblast, and Maritime krai (Zalesskaja et al. 1982; Ganin 1997). Outside Asian Russia: Western Palaearctic.

### ﻿12. Pachymerium pilosum

Taxon classificationAnimaliaGeophilomorphaGeophilidae

(Meinert, 1870) *

DDDF5987-E07B-58E8-AAAA-84DD02B8F0A9

Geophilus pilosus Meinert 1870: 86.
Geophilus pilosus – Stuxberg 1876a: 32; 1876b: 315; Sseliwanoff 1881: 3; 1884: 76; Daday 1889: 146; Attems 1929: 324.
Geophilus (Pachymerium) pilosus – Attems 1903a: 45; 1903b: 257.
Pachymerium pilosum – Muralewicz 1926: 42; Bonato et al. 2016.

#### Type locality.

Russia: Sakhalin oblast: “Sartung, paa Oen Sacolin” (Meinert 1870) = “Sartung” (see Remarks), Sakhalin Isl.

#### Type series.

***Syntypes***: 2 specimens, both sexes. Deposited in NHMD.

#### Diagnosis.

A species of *Pachymerium* with forcipular coxosternite with very short chitin-lines; forcipular tarsungulum without denticle; 45–49 leg-bearing segments; ultimate metasternite narrow, with subparallel lateral edges; all coxal pores sparse from the ventral to the dorsal sides of the coxopleura.

#### Distribution.

Eastern Siberia: Krasnoyarsk krai, Irkutsk oblast (Stuxberg 1876a, 1876b; Sseliwanoff 1881, 1884; Daday 1889). Far East: Sakhalin oblast (Sakhalin Isl.) (Meinert 1870). Outside Asian Russia: no records.

#### Remarks.

Sseliwanoff (1884) reported specimens from “near Baikal”, collected by G. Dybovskiy. According to the catalogue of Chilopoda of the ZMMU, these specimens were collected from the Kultuk (urban-type settlement in the Irkutsk oblast, 51°43'N, 103°40'E).

The assignment of this nominal species to the genus *Pachymerium* is only tentative (Bonato et al. 2016), and is suggested only by the following few characters described by Meinert (1870): head ~ 1.2× as long as wide, forcipular coxosternite very broad and with two anterior denticles, forcipules surpassing the anterior margin of the head, coxopleura elongate and with dense coxal pores not only on the ventral side but also on the lateral and dorsal ones, ultimate legs slightly longer than penultimate legs and bearing a claw.

The current identity and position of the locality “Sartung” (indicated by Meinert 1870) is uncertain.

### ﻿Genus Strigamia

Taxon classificationAnimaliaGeophilomorphaGeophilidae

Gray, 1843

5B351D96-F44F-5C89-82A0-21D236FF2866

#### Diagnosis.

Geophilids with head slightly wider or as wide as long; clypeal areas absent; labrum without obviously distinct lateral parts; second maxillary coxosternite medially long and sclerotized, without both statuminia and anterior inner processes; second maxillary pretarsus relatively small, claw-like; forcipular tergite approximately as wide as the subsequent tergite, covering pleurites almost completely; forcipular coxosternite distinctly shorter than wide, without both anterior denticles and chitin-lines, with coxopleural sutures distinctly diverging also in their anterior half; forcipular trochanteroprefemur relatively stout, without denticle; forcipular tarsungulum with a relatively large basal denticle; trunk sternites without “carpophagus” structures; ventral pore-fields present, at least two paired ovoid posterior fields on each metasternite; coxal pores only on the ventral side of coxopleura, denser near the metasternite; leg of the ultimate pair usually with claw-like pretarsus. See Table 5.

**Table 5.** Differences between species of the genus *Strigamia* Gray, 1843 known from Asian Russia.

Species	Characters							
	Head	Forcipular tarsungula			Anterior sternites	Number of leg-bearing segments	Ultimate leg-bearing segment	
	Clypeal setae	Surpassing the anterior margin of the head	Margins of intermediate part	Size of basal denticle	Mid-longitudinal sclerotized stripe		Intercalary pleurites	Metasternite
S.cf.acuminata (Leach, 1815)	three groups	no	variable	short	no	37–43	no	ca as long as wide
*S.alokosternum* (Attems, 1927)	?	no	subparallel	large	yes	51–67	yes	distinctly wider than long
*S.hirsutipes* (Attems, 1927)	?	no	gradually converging	short	?	39–53	yes	longer than wide
*S.pusilla* (Sseliwanoff, 1884)	?	no	gradually converging	short	no	33–39	yes	as long as wide or longer than wide
*S.sacolinensis* (Meinert, 1870)	?	yes	?	?	no	43–47	?	?
*S.sibirica* (Sseliwanoff, 1881)	?	no	?	?	?	33–35	?	?
*S.sulcata* (Sseliwanoff, 1881)	?	no	?	?	?	41–43	?	?
S.cf.transsilvanica (Verhoeff, 1928)	entire band	no	gradually converging	large	no	43–57	no	ca as long as wide

### ﻿13. Strigamia cf. acuminata

Taxon classificationAnimaliaGeophilomorphaGeophilidae

(Leach, 1815)

DA2B4E9F-2176-5B8E-AACD-DD76B0A8F121

Scolioplanes acuminatus – Sseliwanoff 1881: 15; 1884: 92; Verhoeff 1928: 278; Attems 1929: 222; Takakuwa 1933: 133; 1938: 241; 1940: 124; Shinohara 1972: 66; Kurcheva 1977: 46.
Strigamia acuminata – Ganin 1997: 105, 112, 116, 129, 134, 141; Barber 2009: 74; Bonato et al. 2012: 9; 2023: 11; Volkova 2016: 675.

#### Type localities.

United Kingdom: “Roborough Down near Plymouth” and “Battersea fields” (Leach 1815).

#### Diagnosis.

A species of *Strigamia* with clypeal setae arranged in an intermediate and two lateral groups; forcipular tarsungula not surpassing the anterior margin of the head; basal denticle of forcipular tarsungulum relatively short and with straight converging margins; 37–43 leg-bearing segments; metasternites of the anterior part of the trunk without a mid-longitudinal sclerotized stripe; ultimate leg-bearing segment with pleuropretergite entire, i.e., without distinct intercalary pleurites, and metasternite approximately as long as wide.

#### Distribution.

Far East: Amur oblast and Khabarovsk krai (Ganin 1997), Sakhalin oblast (Kuril Islands) (Takakuwa 1933; Kurcheva 1977). Outside Asian Russia: Europe (Barber 2009; Bonato et al. 2012, 2023; Volkova 2016).

#### Remarks.

All records from Russian Far East need confirmation, as are the records from Japan and the Western part of North America, because of probable confusion with other species including *S.chionophila* Wood, 1862 (Bonato et al. 2012).

### ﻿14. Strigamia alokosternum

Taxon classificationAnimaliaGeophilomorphaGeophilidae

(Attems, 1927)

29788A6E-FC81-55A8-884A-F9DF4060CCAB

Scolioplanes alokosternum Attems 1927: 294.
Scolioplanes alokosternum – Attems 1929: 223; Takakuwa 1938: 243; 1940: 128; Kurcheva 1977: 45.
Strigamia alokosternum – Murakami 1993: 105; Bonato et al. 2012: 9.

#### Type localities.

Japan: “Yamanaka, Suruga” and “Bukenji” (Attems 1927).

#### Type series.

***Syntypes***: 2 specimens, both sexes. Deposited in NHMW (Ilie et al. 2009).

#### Diagnosis.

A species of *Strigamia* with forcipular tarsungula not surpassing the anterior margin of the head; basal denticle of forcipular tarsungulum relatively large; internal and external margins of forcipular tarsungulum subparallel in their intermediate part; 51–67 leg-bearing segments; metasternites of the anterior part of the trunk with a mid-longitudinal sclerotized stripe; ultimate leg-bearing segment with distinct intercalary pleurites, and metasternite distinctly wider than long.

#### Distribution.

Far East: Sakhalin oblast (Sakhalin Isl.) (Kurcheva 1977). Outside Asian Russia: Korean Peninsula and Japan (Bonato et al. 2012).

### ﻿15. Strigamia hirsutipes

Taxon classificationAnimaliaGeophilomorphaGeophilidae

(Attems, 1927) *

4D88EAF6-C765-57ED-831F-DCAFA8BCE704

Scolioplanes hirsutipes Attems 1927: 293.
Scolioplanes hirsutipes – Attems 1929: 222; Takakuwa 1938: 243; 1940: 127; Ghilarov and Perel 1973: 46; Kurcheva 1977: 45.
Strigamia hirsutipes – Ganin 1997: 124, 126, 128; Bonato et al. 2012: 15.

#### Type localities.

Japan: “Kanagava”, “Yamanaka (Suruga)”, “Bukengi”, and Negishi” (Attems 1927).

#### Type series.

***Syntypes***: ca 42 specimens, both sexes. Deposited in NHMW (Ilie et al. 2009).

#### Diagnosis.

A species of *Strigamia* with forcipular tarsungula not surpassing the anterior margin of the head; basal denticle of forcipular tarsungulum relatively short; internal and external margins of forcipular tarsungulum gradually converging through the entire length; 39–53 leg-bearing segments; ultimate leg-bearing segment with distinct intercalary pleurites and metasternite longer than wide.

#### Distribution.

Far East: Maritime krai, Sakhalin oblast (Sakhalin Isl.) (Ghilarov and Perel 1973; Kurcheva 1977; Ganin 1997). Outside Asian Russia: Japan (Attems 1927; Bonato et al. 2012).

#### Remarks.

The taxonomic distinction of this nominal species from *S.sacolinensis* is uncertain.

Records from South-Eastern Asia are doubtful (Bonato et al. 2012).

### ﻿16. Strigamia pusilla

Taxon classificationAnimaliaGeophilomorphaGeophilidae

(Sseliwanoff, 1884)

480CBF03-BF3D-547B-99CF-143BCCD0FAFD

Scolioplanes pusillus Sseliwanoff 1884: 92.
Scolioplanes pusillus – Attems 1929: 224.
Scolioplanes perkeo Verhoeff 1935: 19 (synonymy by Dobroruka 1955: 202).
Scolioplanes pseudopusillus Loksa 1962: 857 (synonymy by Zalesskaja et al. 1982: 190).
Strigamia pusillus – Zalesskaja et al. 1982: 189; Volkova 2016: 675.
Strigamia pusilla – Farzalieva 2008: 64; Bonato et al. 2012: 18; Tuf and Kupka 2015: 110; Poloczek et al. 2016: 117; Tuf and Tajovsky 2016: 47; Nefediev et al. 2017c: 223; 2018: 237; Nefediev 2019: 25; Dyachkov and Farzalieva 2023: 229.

#### Type locality.

Russia: Moscow oblast: “Zaraysk, Ryazanskoy Gubernii” (Sseliwanoff 1884) = Zaraysk city, 54°45'N, 38°53'E.

#### Type series.

***Syntypes***: 11 specimens, including 4 males and 7 females. Deposited in ZISP.

#### Diagnosis.

A species of *Strigamia* with forcipular tarsungula not surpassing the anterior margin of the head; basal denticle of forcipular tarsungulum relatively short, pointed, and with straight outlines; internal and external margins of forcipular tarsungulum gradually converging through the entire length; 33–39 leg-bearing segments; metasternites of the anterior part of the trunk without a mid-longitudinal sclerotized stripe; ultimate leg-bearing segment with distinct intercalary pleurites and metasternite as long as wide or longer than wide.

#### Distribution.

Western Siberia: Sverdlovsk oblast (Zalesskaja et al. 1982; Farzalieva 2008), Altai krai (Nefediev et al. 2018; Nefediev 2019), and Republic of Altai (Nefediev et al. 2017c). Eastern Siberia: Republic of Sakha (Yakutia) (Nefediev 2019). Outside Asian Russia: westwards to Central Europe (Zalesskaja et al. 1982; Bonato et al. 2012; Tuf and Tajovsky 2016; Volkova 2016), and southwards to Northern Mongolia (Poloczek et al. 2016; Dyachkov and Farzalieva 2023).

### ﻿17. Strigamia sacolinensis

Taxon classificationAnimaliaGeophilomorphaGeophilidae

(Meinert, 1870) *

26D59740-B275-58CD-AAD1-C9878830B331

Scolioplanes sacolinensis Meinert 1870: 53.
Scolioplanes sacolinensis – Sseliwanoff 1881: 16; 1884: 93; Attems 1903a: 46; 1903b: 267; 1929: 224; Molodova 1973: 67; Kurcheva 1977: 46.
Strigamia sacolinensis – Ganin 1997: 134; Bonato et al. 2012: 18.

#### Type locality.

Russia: Sakhalin oblast: “Sartung paa Oen Sacalin” (Meinert 1870) = “Sartung” (see Remarks), Sakhalin Isl.

#### Type series.

***Holotype***: female. Deposited in NHMD.

#### Diagnosis.

A species of *Strigamia* with forcipular tarsungula surpassing the anterior margin of the head; 43–47 leg-bearing segments; metasternites of the anterior part of the trunk without a mid-longitudinal sclerotized stripe.

#### Distribution.

Far East: Khabarovsk krai (Sseliwanoff 1881, 1884) and Sakhalin oblast (Sakhalin Isl.) (Meinert 1870; Molodova 1973; Kurcheva 1977; Ganin 1997). Outside Asian Russia: no records.

#### Remarks.

Bonato et al. (2012) suggested the putative projection of the forcipules in front of the anterior margin of the head can be due to some post-mortem displacement of the head with respect to the trunk. Based on the incomplete description provided by Meinert (1870), this nominal species could be a senior synonym of either *S.hirsutipes* or *S.japonica* (Verhoeff, 1935).

The current identity and position of the locality “Sartung” (indicated by Meinert 1870) is uncertain.

### ﻿18. Strigamia sibirica

Taxon classificationAnimaliaGeophilomorphaGeophilidae

(Sseliwanoff, 1881) *

ACF28ECE-E264-5519-8F46-465A2E844297

Scolioplanes sibiricus Sseliwanoff 1881: 16.
Scolioplanes sibiricus – Sseliwanoff 1884: 94; Attems 1903a: 46; 1903b: 268; 1929: 224.
Linotaenia sibirica – Cook 1896: 866.
Strigamia sibirica – Bonato et al. 2012: 18.

#### Type locality.

Russia: Zabaykalsky krai: “Yablonoviy Khrebet” (Sseliwanoff 1881) = Yablonoviy Mt. Range, ca 52°2'N, 113°35'E.

#### Type series.

***Syntypes***: 4 specimens, including 1 male and 3 females. Deposited in ZISP.

#### Diagnosis.

A species of *Strigamia* with forcipular tarsungula not surpassing the anterior margin of the head; 33–35 leg-bearing segments.

#### Distribution.

Eastern Siberia: Zabaykalsky krai (Sseliwanoff 1881). Outside Asian Russia: no records.

#### Remarks.

The distinction between this nominal species and *S.pusilla* is unclear (Bonato et al. 2012).

### ﻿19. Strigamia sulcata

Taxon classificationAnimaliaGeophilomorphaGeophilidae

(Sseliwanoff, 1881) *

26BA24F1-D7EA-5F8A-9355-64572C3E2AC8

Scolioplanes sulcatus Sseliwanoff 1881: 17.
Scolioplanes sulcatus – Sseliwanoff 1884: 95; Attems 1903a: 46; 1903b: 267; 1929: 224.
Linotaenia sulcata – Cook 1896: 866.
Strigamia sulcatus – Ganin 1997: 114, 141.
Strigamia sulcata – Bonato et al. 2012: 19.

#### Type locality.

Russia: Khabarovsk krai: “Nikolaevsk-na-Amure” (Sseliwanoff 1881) = Nikolayevsk-on-Amur city, 53°8'N, 140°42'E.

#### Type series.

***Syntypes***: 3 specimens, including 2 males and 1 female. Deposited in ZISP.

#### Diagnosis.

A species of *Strigamia* with forcipular tarsungula not surpassing the anterior margin of the head; 41–43 leg-bearing segments.

#### Distribution.

Far East: Khabarovsk krai (Sseliwanoff 1881, 1884; Ganin 1997). Outside Asian Russia: no records.

#### Remarks.

The distinction between this nominal species and many other congeneric species is unclear (Bonato et al. 2012).

### ﻿20. Strigamia cf. transsilvanica

Taxon classificationAnimaliaGeophilomorphaGeophilidae

(Verhoeff, 1928)

BAEB53CD-ABA5-5218-8DA5-B7C177FCC1B1

Scolioplanes transsilvanicus Verhoeff 1928: 278.
Scolioplanes transsilvanicus – Takakuwa 1938: 240; 1940: 123; Kurcheva 1977: 45.
Strigamia transsilvanicus – Ganin 1997: 121, 126.
Strigamia transsilvanica – Bonato et al. 2012: 19; 2023: 17.
Strigamia cf. transsilvanica – Zuev and Evsyukov 2016: 425; Nefediev et al. 2018: 237; Dyachkov 2018b: 255; Dyachkov et al. 2022: 75; Dyachkov and Zuev 2023: 159.

#### Type localities.

Romania: “Hermannstadt” = Sibiu. Slovenia: “Gottschee” = Kocevje. Austria: “Ostalpen” = Eastern Alps; “Hermagor”; “Arlberg”. Italy: “Schneelagern am Schlüsseljoch beim Brenner” = Colle della Chiave, near Brennero. Slovakia: “Tatra-Höhlenhain” = Tatranska Kotlina. Germany: “Titisee”. Switzerland: “Pilatus Kulm” (Verhoeff 1928).

#### Diagnosis.

A species of *Strigamia* with clypeal setae uniformly spaced in a continuous array, without recognizable gaps between intermediate and lateral groups of setae; forcipular tarsungula not surpassing the anterior margin of the head; basal denticle of forcipular tarsungulum relatively large and with distinctly curved outlines; internal and external margins of forcipular tarsungulum gradually converging through the entire length; 43–57 leg-bearing segments; metasternites of the anterior part of the trunk without a mid-longitudinal sclerotized stripe; ultimate leg-bearing segment with pleuropretergite entire, i.e., without distinct intercalary pleurites, and metasternite approximately as long as wide.

#### Distribution.

Western Siberia: Altai krai (Nefediev et al. 2018). Far East: Maritime krai, Sakhalin oblast (Sakhalin Isl.) (Kurcheva 1977; Ganin 1997). Outside Asian Russia: westwards to Central Europe (Iorio 2005; Reip and Voigtländer 2009).

#### Remarks.

*Strigamiatranssilvanica* belongs to a species complex whose taxonomy is only partially resolved (Bonato et al. 2023).

Bonato et al. (2012) suggested that the records from Russian Far East are probably due to misidentification of a different species. Nefediev et al. (2018) suggested the presence of a possible undescribed species similar in morphology to *S.transsilvanica* from Western Siberia, so that also the presence of *S.transsilvanica* in Western Siberia is doubtful. Doubtful are also the records from European Russia and Caucasus (Zuev and Evsyukov 2016; Dyachkov et al. 2022; Dyachkov and Zuev 2023), Eastern Kazakhstan (Dyachkov 2018b), Japan, and Taiwan (Bonato et al. 2012).

**Family Mecistocephalidae Bollman, 1893**

### ﻿Genus Agnostrup

Taxon classificationAnimaliaGeophilomorphaMecistocephalidae

Foddai, Bonato, Pereira & Minelli, 2003

4BE41A9A-6C2F-598B-8D2F-02B785F6B553

#### Diagnosis.

Mecistocephalids with head moderately longer than wide; two clypeal plagulae, separated by a mid-longitudinal areolate stripe and extending to the lateral margins of the clypeus; cephalic pleurites without both spiculum and setae; first maxillary coxosternite medially divided by a sulcus, without antero-lateral corners; second maxillary coxosternite medially undivided, with the grooves from the metameric pores reaching the posterior corners; second maxillary telopodites relatively small, not distinctly overreaching the first maxillary telopodites, without pretarsus; forcipular tergite distinctly wider than long, without a distinct mid-longitudinal sulcus; forcipular trochanteroprefemur with only a distal denticle, tarsungulum with a basal denticle; invariably 41 leg-bearing segments; sternites with non-furcate mid-longitudinal sulcus and without pore-fields; legs of the ultimate pair ending with a short spine. See Table 6.

**Table 6.** Differences between members of the family Mecistocephalidae Bollman, 1893 known from Asian Russia.

Species	Characters						
	Clypeal plagulae	First maxillary coxosternite: mid-longitudinal sulcus	Second maxillary telopodites surpassing first maxillary telopodites	Second maxillary pretarsus	Denticle on forcipular tarsingulum	Denticles on forcipular intermediate articles	Leg-bearing segments
*Agnostrupstriganovae* (Titova, 1975)	two, extending to lateral margins of clypeus	yes	no	no	yes	small bulges	41
*Arrupdentatus* (Takakuwa, 1934)	two, not extending to lateral margins of clypeus	no	no	yes	yes	large on tibia	41
*Arrupmamaevi* (Titova, 1975)	two, not extending to lateral margins of clypeus	no	no	no	yes	small bulges	41
*Tygarrupjavanicus* Attems, 1929	single, extending to lateral margins of clypeus	yes	yes	yes	no	tibia with denticle	45

### ﻿21. Agnostrup striganovae

Taxon classificationAnimaliaGeophilomorphaMecistocephalidae

(Titova, 1975)

74DFB541-2D54-532E-BA15-57CAF31148DC

Krateraspis striganovae Titova 1975: 40.
Krateraspis striganovae – Markelov and Mineeva 1981: 130; Ganin 1997: 124; 2011: 341.
Agnostrup striganovae – Foddai et al. 2003: 1254.

#### Type locality.

Russia: Maritime krai: “Sudzuhinsky Zapovednik, Tachingauz” (Titova 1975) = Lazovsky Nature Reserve, Tachingauz bay, ca 43°1'N, 134°8'E.

#### Type series.

***Holotype***: male. Deposited in ZMMU (S. Golovatch and A. Schileyko, pers. comm., 13.II.2023 and XII.2023).

#### Diagnosis.

An *Agnostrup* species with body length reaching ≥ 3 cm; clypeal plagulae with an irregular anterior margin and slightly smaller than the areolate part of the clypeus; many setae near the anterior margin of plagulae and on the center of the areolate part of the clypeus; forcipular trochanteroprefemur 1.3× as long as wide, both forcipular femur and tibia with small bulges.

#### Distribution.

Far East: Maritime krai (Titova 1975; Markelov and Mineeva 1981; Ganin 1997). Outside Asian Russia: no records.

### ﻿Genus Arrup

Taxon classificationAnimaliaGeophilomorphaMecistocephalidae

Chamberlin, 1912

1584ADBC-AF73-5419-A944-F205EC14B7A6

#### Diagnosis.

Mecistocephalids with head moderately longer than wide; two clypeal plagulae, separated by a mid-longitudinal areolate stripe and not extending to the lateral margins of the clypeus; cephalic pleurites without both spiculum and setae; first maxillary coxosternite medially undivided, without antero-lateral corners; second maxillary coxosternite medially undivided, with the grooves from the metameric pores reaching the posterior corners; second maxillary telopodites relatively small, not distinctly overreaching the first maxillary telopodites, usually without pretarsus; forcipular tergite distinctly wider than long, without a distinct mid-longitudinal sulcus; forcipular trochanteroprefemur with only a distal denticle, tarsungulum with a basal denticle; invariably 41 leg-bearing segments; sternites with non-furcate mid-longitudinal sulcus and without pore-fields; legs of the ultimate pair ending with a short spine. See Table 6.

### ﻿22. Arrup dentatus

Taxon classificationAnimaliaGeophilomorphaMecistocephalidae

(Takakuwa, 1934)

200E15CA-E4E5-55A6-8212-098D4E254F20

Prolamnonyx dentatus Takakuwa 1934a: 707 (see Remarks).
Prolamnonyx dentatus – Takakuwa 1934b: 359; 1934c: 883.
Prolamnonyx dentatus – Shinohara 1972: 66; Titova 1975: 45; Ganin 1997: 134.
Arrup dentatus – Crabill 1964: 166; Foddai et al. 2003: 1261; Uliana et al. 2007: 13.

#### Type locality.

Japan: Hokkaido: “Zyôzankei (bei Sapporo)” (Takakuwa 1934b).

#### Type series.

Unknown number of specimens, possibly lost (Jonishi and Nakano 2022). Depositary unknown.

#### Diagnosis.

An *Arrup* species with body reaching ≥ 2 cm; second maxillary pretarsi present (see Remarks); forcipular trochanteroprefemur with a large distal denticle, tibia with large denticle, tarsungulum with pointed basal denticle.

#### Distribution.

Far East: Maritime krai, Sakhalin oblast (Sakhalin and Kuril Islands: Shikotan) (Titova 1975; Ganin 1997). Outside Asian Russia: Japan (Takakuwa 1934b; Shinohara 1972).

#### Remarks.

The name *Prolamnonyxdentatus* was validly introduced by Takakuwa (1934a) in a key; specimens were later described in more detail by Takakuwa (1934b, 1934c). Uliana et al. (2007) described the presence of second maxillary pretarsi in this species, according to the original description and Titova (1975), it is absent in this species.

### ﻿23. Arrup mamaevi

Taxon classificationAnimaliaGeophilomorphaMecistocephalidae

(Titova, 1975)

33F6C369-0D8F-51E2-88A5-81DBAC7305FE

Prolamnonyx mamaevi Titova 1975: 44.
Prolamnonyx holstii – Titova 1969: 165 (see Remarks).
Prolamnonyx mamaevi – Ganin 1997: 121.
Arrup mamaevi – Foddai et al. 2003: 1262.

#### Type locality.

Russia: Maritime krai: “Primorsky Kray, zapovednik Kedrovaya Pad”, Kedrovaya Pad Nature Reserve, ca 43°05'N, 131°30'E, (Titova 1975).

#### Type series.

***Holotype***: female. Deposited in ZMMU (S. Golovatch and A. Schileyko, pers. comm., 13.II.2023 and XII.2023).

#### Diagnosis.

An *Arrup* species with body length reaching ≥ 3 cm; second maxillary pretarsi absent; forcipular trochanteroprefemur with a large distal denticle, both femur and tibia with small bulges, tarsungulum with pointed basal denticle.

#### Distribution.

Far East: Maritime krai (Titova 1975; Ganin 1997). Outside Asian Russia: no records.

#### Remarks.

The holotype had been previously assigned to *Prolamnonyxholstii* (Pocock, 1895) by Titova (1969).

### ﻿Genus Tygarrup

Taxon classificationAnimaliaGeophilomorphaMecistocephalidae

Chamberlin, 1914

033E3F77-650E-5839-8C96-E747524FEE15

#### Diagnosis.

Mecistocephalids with head distinctly longer than wide; clypeus with an entire plagula, without mid-longitudinal areolate stripe and extending to the lateral margins of the clypeus; cephalic pleurites without both spiculum and setae; first maxillary coxosternite medially divided by a sulcus, without antero-lateral corners; second maxillary coxosternite medially undivided, with the grooves from the metameric pores reaching the lateral margins; second maxillary telopodites distinctly overreaching the first maxillary telopodites, with claw-like pretarsus; forcipular tergite only slightly wider than long, without a distinct mid-longitudinal sulcus; forcipular trochanteroprefemur with only a distal denticle, tarsungulum without denticle; invariably 43 or 45 leg-bearing segments; sternites with non-furcate mid-longitudinal sulcus and sometimes with pore-fields; legs of the ultimate pair ending with a short spine. See Table 6.

### ﻿24. Tygarrup javanicus

Taxon classificationAnimaliaGeophilomorphaMecistocephalidae

Attems, 1929

BE19F3A4-1895-575B-BAA4-E024C04694BC

Tygarrup javanicus Attems 1929: 152.
Tygarrup javanicus – Bonato et al. 2004; Nefediev 2019: 24; Tuf et al. 2018: 560; Damasiewicz and Leśniewska 2020: 52; Gilgado et al. 2022: 92.

#### Type localities.

Indonesia: Java: “Buitenzorg”, “Tjibodas” and “Tjompea” (Attems 1929).

#### Diagnosis.

A *Tygarrup* species with body length ≤ 2 cm; no distinct dark patches along the body; second maxillary pretarsus with a long slender point; both forcipular trochanteroprefemur and tibia with denticles; invariably 45 leg-bearing segments; ventral pore-fields absent in females, present in males; metasternite of the ultimate leg-bearing segment slightly wider than long.

#### Distribution.

Western Siberia: Altai krai (Nefediev 2019). Outside Asian Russia: Southeast Asia and introduced in Europe (e.g., Bonato et al. 2004; Tuf et al. 2018; Damasiewicz and Leśniewska 2020; Gilgado et al. 2022).

#### Remarks.

The species is regarded as an anthropochore introduction in Asian Russia (Nefediev 2019).

**Family Schendylidae Cook, 1896**

### ﻿Genus Escaryus

Taxon classificationAnimaliaGeophilomorphaSchendylidae

Cook & Collins, 1891

92537473-4C61-5CF7-AB70-E2129E2F3A44

#### Diagnosis.

Schendylids with head slightly longer than wide; antennae gradually tapering; labrum with distinct denticles in the intermediate part; first maxillae with lappets; second maxillary pretarsi fringed by two rows of filaments; forcipular tergite narrower than subsequent tergite; ventral pore-fields absent; coxal pores numerous and scattered; legs of the ultimate pair with two tarsal articles and claw-like pretarsus, swollen in adult males and slender in females; gonopods biarticulated in both sexes. See Table 7.

**Table 7.** Differences between species of the genus *Escaryus* Cook & Collins, 1891 known from Asian Russia.

Species	Characters									
	Labrum		First maxillae: pairs of lappets	Denticles on forcipular articles				Leg-bearing segments	Ultimate leg-bearing segment: metasternite: shape, length/width	Anal pores
	Margin	Denticles		Trochanteroprefemur	Femur	Tibia	Tarsungulum			
*E.chadaevae* Titova, 1973	shallow	short and obtuse	1	small bulge	small bulge	large bulge	large denticle	33–35	trapezoid, < 1	absent
*E.chichibuensis* Shinohara, 1955	shallow	short and obtuse	1	small bulge	small bulge	small bulge	no	35–39	trapezoid, ~ 1	present
*E.dentatus* Titova, 1973	shallow	short and obtuse	1	large	small	small	large	37–39	trapezoid, > 1	present
*E.hirsutus* Titova 1973	deep	long and obtuse	1	large bulge	large bulge	large bulge	large bulge	37–39	rectangular, 1.5	present
*E.japonicus* Attems, 1927	shallow	long and obtuse	1	small	small bulge	small bulge	small bulge	43–55	rectangular, 2	present
*E.koreanus* Takakuwa, 1937	shallow	long, middle denticles obtuse, lateral ones pointed	1	small	no	no	small bulge	43–55	rectangular, 2	present
*E.krivolutskiji* Titova, 1973	deep	short and obtuse	1	large bulge	small bulge	large bulge	small bulge	45–49	trapezoid, ~ 1	present
*E.molodovae* Titova, 1973	shallow	short and obtuse	1	small	small	small	large	35	trapezoid, ~ 1	present
*E.perelae* Titova, 1973	shallow	short and obtuse	1	small	no	small	large	39–43	trapezoid, < 1	present
*E.polygonatus* Titova, 1973	deep	short and obtuse	1	small bulge	small bulge	small bulge	no	39	trapezoid, 1.5	present
*E.retusidens* Attems, 1904	deep	long and obtuse	1	small	small	small	small bulge	49–55	trapezoid, ~ 1	absent
*E.sachalinus* Takakuwa, 1935	deep	short and obtuse	?	small	small	small	no	35–39	rectangular, 1.5	present
*E.sibiricus* Cook, 1899	shallow	middle denticles obtuse, lateral ones long and pointed	2	small bulge	no	no	no	49–51	rectangular, 2	absent
*E.vitimicus* Titova, 1973	shallow	long and obtuse	1	small bulge	small bulge	small bulge	small bulge	37	rectangular, 1.5	present

### ﻿25. Escaryus chadaevae

Taxon classificationAnimaliaGeophilomorphaSchendylidae

Titova, 1973

19D3752C-976D-5623-9E84-187008AA40D7

Escaryus chadaevae Titova 1973: 105.
Escaryus chadaevae – Rybalov 2002: 83; Vorobiova et al. 2002: 62; Poloczek et al. 2016: 117; Volkova 2016: 675; Nefediev et al. 2017c: 221; Dyachkov and Farzalieva 2023: 229.

#### Type localities.

Russia: Kemerovo oblast: “Prokopyevsky i Novokuznetsky r-ny” (Titova 1973) = Prokopyevsky, ca 53°53'N, 86°43'E, and Novokuznetsky, ca 53°45'N, 87°07'E, districts.

#### Type series.

***Holotype***: female. Paratypes: 10 specimens, including 5 males and 5 females. Deposited in ZMMU (S. Golovatch and A. Schileyko, pers. comm., 13.II.2023 and XII.2023).

#### Diagnosis.

An *Escaryus* species with body length reaching ≥ 1.5 cm; clypeus without plagulae; labral arc relatively shallow, with denticles short and obtuse; first maxillae with one pair of lappets; forcipular trochanteroprefemur and femur with small bulges, tibia with a large bulge, tarsungulum with a large basal denticle; 33–35 leg-bearing segments; metasternite of the ultimate leg-bearing segment trapezoid, distinctly wider than long; coxal pores only on the ventral side of coxopleura; anal pores absent.

#### Distribution.

Western Siberia: Kemerovo oblast, Republic of Altai (Titova 1973; Nefediev et al. 2017c). Eastern Siberia: Krasnoyarsk krai (Rybalov 2002; Vorobiova et al. 2002). Outside Asian Russia: European Russia (Republic of Bashkortostan) (Titova 1973; Volkova 2016), and Northern Mongolia (Poloczek et al. 2016; Dyachkov and Farzalieva 2023).

### ﻿26. Escaryus chichibuensis

Taxon classificationAnimaliaGeophilomorphaSchendylidae

Shinohara, 1955 *

671C9862-852C-5031-8250-26C17DDDAC7D

Escaryus chichibuensis Shinohara 1955: 59.
Escaryus chichibuensis – Titova 1973: 114; Kurcheva 1977: 45.

#### Type localities.

Japan: Honshu: Saitama Prefecture: “Chichibu (Mt. Kumotori, Kasatori pass, Mt. Kobushi, Kabagoya-ato, Karisaka pass, Jumonji pass, Mt. Shiraiwa, Mt. Mae-Shiraiwa” (Shinohara 1955).

#### Type series.

***Syntypes***: unknown number of specimens, both sexes. Depository unknown.

#### Diagnosis.

An *Escaryus* species with body length reaching ≥ 2 cm; clypeus with small plagulae; labral arc relatively shallow, with denticles short and obtuse; first maxillae with one pair of lappets; forcipular trochanteroprefemur, femur, and tibia with small bulges, tarsungulum without bulge; 35–39 leg-bearing segments; metasternites with relatively sparse setae; metasternite of the ultimate leg-bearing segment trapezoid, approximately as long as wide; coxal pores on both ventral and lateral sides of coxopleura; anal pores present.

#### Distribution.

Far East: Sakhalin oblast (Kuril Islands: Kunashir and Shikotan) (Titova 1973; Kurcheva 1977). Outside Asian Russia: Japan (Shinohara 1955).

### ﻿27. Escaryus dentatus

Taxon classificationAnimaliaGeophilomorphaSchendylidae

Titova, 1973 *

1FF05571-CED9-51B4-B989-E8D8AE9A2C12

Escaryus dentatus Titova 1973: 99.
Escaryus dentatus – Kurcheva 1977: 45; Markelov and Mineeva 1981: 130; Ganin 1997: 121, 124, 126, 128; 2006: 501.

#### Type localities.

Russia: Maritime krai: “Suputinsky zapovednik” = Ussuriysky Nature Reserve, ca 43°40'N, 132°32'E, and “Kedrovaya Pad” (both Titova 1973) = Kedrovaya Pad Nature Reserve, ca 43°05'N, 131°30'E, (see Remarks).

#### Type series.

***Holotype***: female. Paratypes: 11 specimens, including 6 males and 5 females. Deposited in ZMMU (S. Golovatch and A. Schileyko, pers. comm., 13.II.2023 and XII.2023).

#### Diagnosis.

An *Escaryus* species with body length reaching ≥ 2 cm; clypeus without plagulae (polygonal structure poorly visible, but recognizable); labral arc relatively shallow, with denticles short and obtuse; first maxillae with one pair of lappets; forcipular trochanteroprefemur and tarsungulum with large denticles, femur and tibia with small denticles; 37–39 leg-bearing segments; metasternites with relatively sparse setae; metasternite of the ultimate leg-bearing segment trapezoid, slightly longer than wide; coxal pores on both ventral and lateral sides of coxopleura; anal pores present.

#### Distribution.

Far East: Maritime krai (Titova 1973; Kurcheva 1977; Ganin 1997, 2006). Outside Asian Russia: no records.

#### Remarks.

Titova (1973) indicated that the type series was from two localities (“Suputinsky zapovednik” and “Kedrovaya Pad”), but she did not state explicitly which is the locality of the holotype.

### ﻿28. Escaryus hirsutus

Taxon classificationAnimaliaGeophilomorphaSchendylidae

Titova 1973 *

541E8942-5805-5693-B47E-FB12855C4AA7

Escaryus hirsutus Titova 1973: 96.
Escaryus hirsutus – Molodova 1973: 67; Kurcheva 1977: 45; Ganin 1997: 134.

#### Type locality.

Russia: Sakhalin oblast: “O-v Sakhalin, Yuzhno-Sakhalinsk, gora Chekhova” (Titova 1973) = Chekhova Mt., near Yuzhno-Sakhalinsk city, ca 47°00'N, 142°50'E.

#### Type series.

Holotype: female. Paratypes: 10 specimens, including 5 males and 5 females. Deposited in ZMMU (S. Golovatch and A. Schileyko, pers. comm., 13.II.2023 and XII.2023).

#### Diagnosis.

An *Escaryus* species with body length reaching ≥ 2.5 cm; clypeus with large plagulae; labral arc relatively deep, with denticles long and obtuse; first maxillae with one pair of lappets; all forcipular articles with large bulges; 37–39 leg-bearing segments; metasternites with relatively dense setae; metasternite of the ultimate leg-bearing segment almost rectangular, ~ 1.5× as long as wide; coxal pores on both ventral and lateral sides of coxopleura; anal pores present.

#### Distribution.

Far East: Sakhalin oblast (Sakhalin Isl.) (Titova 1973; Molodova 1973; Kurcheva 1977; Ganin 1997). Outside Asian Russia: no records.

### ﻿29. Escaryus japonicus

Taxon classificationAnimaliaGeophilomorphaSchendylidae

Attems, 1927 *

0C851E06-C66A-5E68-B086-66CDCF38DC74

Escaryus japonicus Attems 1927: 299.
Escaryus japonicus – Attems 1929: 96; Byzova and Chadaeva 1965: 333; Shinohara 1972: 66; Titova 1969: 165; 1972: 135; 1973: 113; Ghilarov and Perel 1973: 46; Molodova 1973: 67; Alekseeva 1974: 8; Kurcheva 1977: 45; Markelov and Mineeva 1981: 130; Ganin 1997: 105, 109, 112, 114, 121, 124, 126, 129, 134, 141; 2006: 501; Vorobiova 1999: 33; Farzalieva 2008: 67; Volkova 2016: 676; Dyachkov 2017: 454; Nefediev et al. 2017a: 11; 2017c: 222; Dyachkov and Tuf 2018: 296; Nefediev 2019: 25; Dyachkov and Farzalieva 2023: 229.

#### Type locality.

Japan: Hokkaido: “Todohokhe” (Attems 1927).

#### Type series.

***Syntypes***: 2 specimens, including a male and a juvenile. Deposited in NHMW (Ilie et al. 2009).

#### Diagnosis.

An *Escaryus* species with body length reaching ≥ 4.3 cm; clypeus with large plagulae; labral arc relatively shallow, with denticles long and obtuse; first maxillae with one pair of lappets; forcipular trochanteroprefemur with a small distal denticle, all other articles with very small bulges; 43–55 leg-bearing segments; metasternites with relatively sparse setae; metasternite of the ultimate leg-bearing segment rectangular, ~ 2× as long as wide; coxal pores of similar size, on both ventral and lateral sides of coxopleura; anal pores present.

#### Distribution.

Western Siberia: Sverdlovsk, Chelyabinsk, Tomsk, and Kemerovo oblasts, republics of Altai and Khakassia (Byzova and Chadaeva 1965; Titova 1972, 1973; Farzalieva 2008; Dyachkov 2017; Nefediev et al. 2017a, 2017c; Nefediev 2019). Eastern Siberia: Republic of Buryatia and Krasnoyarsk krai (Alekseeva 1974; Titova 1973; Vorobiova 1999), Magadan oblast (Berman and Leirikh 2019). Far East: Amur oblast, Maritime and Khabarovsk krais, Sakhalin oblast (Sakhalin Isl.) (Titova 1973; Ghilarov and Perel 1973; Molodova 1973; Kurcheva 1977; Markelov and Mineeva 1981; Ganin 1997, 2006). Outside Asian Russia: European Russia (Titova 1973; Farzalieva 2008; Volkova 2016), eastern Kazakhstan (Dyachkov and Tuf 2018), Mongolia (Dyachkov and Farzalieva 2023), Northern China (Takakuwa and Takashima 1949), and Japan (Attems 1927).

#### Remarks.

The record from the Krasnoyarsk krai by Vorobiova (1999) was questioned by Nefediev et al. (2017a).

### ﻿30. Escaryus koreanus

Taxon classificationAnimaliaGeophilomorphaSchendylidae

Takakuwa, 1937 *

C71816E0-2517-590B-B5F0-0F7ECC61766F

Escaryus koreanus Takakuwa 1937a: 297.
Escaryus koreanus – Takakuwa 1940: 39; Titova 1972: 135; 1973: 112; Ghilarov and Perel 1973: 46; Kurcheva 1977: 45; Ganin 1997: 105, 109, 112, 114, 121, 124, 126, 128; 2006: 501; Rybalov 2002: 83; Vorobiova et al. 2002: 62; Nefediev et al. 2017a: 11; 2017c: 222; 2018: 238; Dyachkov 2017: 454; Dyachkov and Tuf 2018: 296; Nefediev 2019: 26.

#### Type locality.

North Korea: “Husenzan” (Takakuwa 1937a).

#### Type series.

***Syntypes***: unknown number of specimens, both sexes. Depository unknown.

#### Diagnosis.

An *Escaryus* species with body length reaching ≥ 6.5 cm; clypeus with large plagulae; labral arc relatively shallow, with long denticles, the middle denticles obtuse, the lateral ones pointed; first maxillae with one pair of lappets; forcipular trochanteroprefemur with an small obtuse denticle, femur and tibia without denticles, tarsungulum with a small basal bulge; 43–55 leg-bearing segments; metasternites with relatively dense setae; metasternite of the ultimate leg-bearing segment rectangular, ~ 2× as long as wide; coxal pores of different size, on both ventral and lateral sides of coxopleura, including a pair of much larger pores on each coxopleuron; anal pores present.

#### Distribution.

Western Siberia: Altai krai, republics of Altai and Khakassia, Kemerovo, Novosibirsk, and Tomsk oblasts (Titova 1972, 1973; Dyachkov 2017; Nefediev et al. 2017a, 2017c, 2018; Nefediev 2019). Eastern Siberia: Krasnoyarsk krai, Irkutskaya oblast (Rybalov 2002; Vorobiova et al. 2002; Nefediev 2019). Far East: Maritime and Khabarovsk krais, Amur Oblast (Titova 1972, 1973; Ghilarov and Perel 1973; Kurcheva 1977; Ganin 1997). Outside Asian Russia: Eastern Kazakhstan (Dyachkov and Tuf 2018), Japan, and North Korea (Takakuwa 1937a, 1940).

#### Remarks.

Titova (1973: 113) suggested that *E.koreanus* can be a junior synonym of *E.sibiricus* Cook, 1899.

### ﻿31. Escaryus krivolutskiji

Taxon classificationAnimaliaGeophilomorphaSchendylidae

Titova, 1973 *

E91A37D7-4E3D-5D47-A5E9-08A0ADB3B47A

Escaryus krivolutskiji Titova 1973: 102.
Escaryus krivolutskiji – Kurcheva 1977: 45; Markelov and Mineeva 1981: 129; Ganin 1997: 121, 124, 126, 128.

#### Type localities.

Russia: Maritime krai: “Suputinsky zapovednik” = Ussuriysky Nature Reserve, ca 43°40'N, 132°32'E, and “Kangauz” = Anisimovka Village, ca 43°10'N, 132°47'E and “Kedrovaya Pad” = Kedrovaya Pad Nature Reserve, ca 43°05'N, 131°30'E, (all Titova 1973) (see Remarks).

#### Type series.

***Holotype***: male. Paratypes: 10 specimens, including 5 males and 5 females. Deposited in ZMMU (S. Golovatch and A. Schileyko, pers. comm., 13.II.2023 and XII.2023).

#### Diagnosis.

An *Escaryus* species with body length reaching ≥ 2 cm; clypeus without plagulae; labral arc relatively deep, with denticles short and obtuse; first maxillae with one pair of lappets; forcipular trochanteroprefemur and tibia with large bulges, femur and tarsungulum with small bulges; 45–49 leg-bearing segments; metasternite of the ultimate leg-bearing segment trapezoid, approximately as long as wide; coxal pores on both ventral and lateral sides of coxopleura, the pair of largest pores close to inner edge of coxopleura; anal pores present.

#### Distribution.

Far East: Maritime krai (Titova 1973; Kurcheva 1977; Markelov and Mineeva 1981; Ganin 1997, 2006). Outside Asian Russia: no records.

#### Remarks.

Titova (1973) indicated that the type series was from three localities (“Suputinsky zapovednik”, “Kangauz”, and “Kedrovaya Pad”), but she did not state explicitly which is the locality of the holotype.

### ﻿32. Escaryus molodovae

Taxon classificationAnimaliaGeophilomorphaSchendylidae

Titova, 1973 *

C8BDE4EF-3A8A-5095-88EC-A88C3EF8A6B9

Escaryus molodovae Titova 1973: 95.
Escaryus molodovae – Molodova 1973: 67; Kurcheva 1977: 45; Ganin 1997: 134.

#### Type locality.

Russia: Sakhalin oblast: “O-v Sakhalin, Yuzhno-Sakhalinsk, gora Chekhova” (Titova 1973) = Chekhova Mt., near Yuzhno-Sakhalinsk city, ca 47°0'N, 142°50'E.

#### Type series.

***Holotype***: male. Paratypes: 8 specimens, including 5 males and 3 females. Deposited in ZMMU (S. Golovatch and A. Schileyko, pers. comm., 13.II.2023 and XII.2023).

#### Diagnosis.

An *Escaryus* species with body length reaching ≥ 1.4 cm: clypeus with small plagulae; labral arc relatively shallow, with denticles short and obtuse; first maxillae with one pair of lappets; forcipular trochanteroprefemur, femur and tibia with small denticles; forcipular tarsungulum with a large, pointed basal denticle; 35 leg-bearing segments; metasternites with relatively sparse setae; metasternite of the ultimate leg-bearing segment trapezoid, approximately as long as wide; coxal pores only on the ventral side of coxopleura; anal pores present.

#### Distribution.

Far East: Sakhalin oblast (Sakhalin Isl.) (Titova 1973; Molodova 1973; Kurcheva 1977; Ganin 1997). Outside Asian Russia: no records.

### ﻿33. Escaryus perelae

Taxon classificationAnimaliaGeophilomorphaSchendylidae

Titova, 1973 *

65B77E7E-07D6-53DE-A977-0C1675C31BC1

Escaryus perelae Titova 1973: 101.
Escaryus perelae – Kurcheva 1977: 45; Markelov and Mineeva 1981: 129; Ganin 1997: 105, 109, 112, 121, 124, 126, 128; 2006: 501.

#### Type localities.

Russia: Maritime krai: “Suputinsky zapovednik” = Ussuriysky Nature Reserve, ca 43°40'N, 132°32'E, and “Rayon r. Sinancha” = near Cheremukhovaya River, inflow of Dzhigitovka River, ca 44°50'N, 136°07'E (both Titova 1973) (see Remarks).

#### Type series.

***Holotype***: female. Paratypes: 6 specimens, including 2 males and 4 females. Deposited in ZMMU (S. Golovatch and A. Schileyko, pers. comm., 13.II.2023 and XII.2023).

#### Diagnosis.

An *Escaryus* species with body length reaching ≥ 2 cm; clypeus with small plagulae; labral arc relatively shallow, with denticles short and obtuse; first maxillae with one pair of lappets; forcipular trochanteroprefemur and tibia with small denticles, femur without denticle, tarsungulum with a large basal denticle; 39–43 leg-bearing segments; metasternite of the ultimate leg-bearing segment trapezoid, distinctly wider than long; most of coxal pores on the ventral side of coxopleura, mostly close to metasternite; anal pores present.

#### Distribution.

Far East: Amur oblast, Maritime and Khabarovsk krais (Titova 1973; Kurcheva 1977; Markelov and Mineeva 1981; Ganin 1997, 2006). Outside Asian Russia: no records.

#### Remarks.

Titova (1973) indicated that the type series was from two localities (“Suputinsky zapovednik” and “Rayon r. Sinancha”), but she did not state explicitly which is the locality of the holotype.

### ﻿34. Escaryus polygonatus

Taxon classificationAnimaliaGeophilomorphaSchendylidae

Titova, 1973 *

6BE4FA1B-44F2-5CD7-83EF-1840073AE535

Escaryus polygonatus Titova 1973: 98.
Escaryus polygonatus – Kurcheva 1977: 45; Markelov and Mineeva 1981: 130; Ganin 1997: 121, 124, 126, 128; 2006: 501.

#### Type localities.

Russia: Maritime krai: “Suputinsky zapovednik” = Ussuriysky Nature Reserve, ca 43°40'N, 132°32'E, and “Kedrovaya Pad” = Kedrovaya Pad Nature Reserve, ca 43°05'N, 131°30'E, (both Titova 1973).

#### Type series.

***Holotype***: male. Paratypes: 17 specimens, including 9 males and 8 females. Deposited in ZMMU (S. Golovatch and A. Schileyko, pers. comm., 13.II.2023 and XII.2023).

#### Diagnosis.

An *Escaryus* species with body length reaching ≥ 2.8 cm; clypeus with small plagulae; labral arc relatively deep, with denticles short and obtuse; first maxillae with one pair of lappets; forcipular trochanteroprefemur, femur, and tibia with small bulges; forcipular tarsungulum without denticle; 39 leg-bearing segments; metasternites with relatively sparse setae; metasternite of the ultimate leg-bearing segment trapezoid, ~ 1.5× as long as wide; coxal pores of different size, on both ventral and lateral sides of coxopleura; anal pores present.

#### Distribution.

Far East: Maritime krai (Titova 1973; Kurcheva 1977; Markelov and Mineeva 1981; Ganin 1997). Outside Asian Russia: no records.

#### Remarks.

Titova (1973) indicated that the type series was from two localities (“Suputinsky zapovednik” and “Kedrovaya Pad”), but she did not state explicitly which is the locality of the holotype.

### ﻿35. Escaryus retusidens

Taxon classificationAnimaliaGeophilomorphaSchendylidae

Attems, 1904

C4E48964-6480-50C1-BC78-CC928941CF3F

Escaryus retusidens Attems 1904: 121.
Escaryus retusidens – Attems 1929: 96; Titova 1972: 135, 116; 1973: 110; Volkova 2016: 675; Zuev 2016: 33; Nefediev et al. 2017a: 11; 2017c: 222; 2018: 239; Dyachkov and Tuf 2018: 295; Nefediev 2019: 27; Dyachkov 2022: 25.

#### Type locality.

Kyrgyzstan: Issyk-Kul Region: “Przewalsk” (Attems 1904) = Karakol.

#### Type series.

***Syntypes***: 4 specimens, including 2 males and 2 females. Deposited in NHMW (Ilie et al. 2009).

#### Diagnosis.

An *Escaryus* species with body length reaching ≥ 4 cm; clypeus with small plagulae; labral arc relatively deep, with denticles long and obtuse; first maxillae with one pair of lappets; forcipular trochanteroprefemur, femur, and tibia with small denticles, tarsungulum with a small bulge; 49–55 leg-bearing segments; metasternites with relatively sparse setae; metasternite of the ultimate leg-bearing segment trapezoid, approximately as long as wide; coxal pores on both ventral and lateral sides of coxopleura; anal pores absent.

#### Distribution.

Western Siberia: Altai krai, Republic of Altai, and Kemerovo oblast (Nefediev et al. 2017a, 2017c, 2018; Nefediev 2019). Possibly Far East (Titova 1972, 1973; see Remarks). Outside Asian Russia: westwards to Moldova (Titova 1973; Volkova 2016; Zuev 2016; Zuev and Evsyukov 2016); southwards to Kazakhstan and Kyrgyzstan (Attems 1904; Titova 1972, 1973; Dyachkov and Tuf 2018; Dyachkov 2022).

#### Remarks.

A total of 19 males and 26 females collected from Trans-Ili Alatau (Almaty Region of Kazakhstan) were indicated by Titova (1973: 110) as lectotypes, but this action is not valid, as the syntypes still exist (Ilie et al. 2009).

Titova (1972: 135; 1973: 116) wrote “... it is possible to distinguish territories from Cisamuria to Kuzbass and Altai, where 4 species are spread: *E.koreanus*, *E.japonicus*, *E.retusidens*, and *E.chadaevae*”. However, she did not mention material from this area when she listed the studied material of *E.retusidens* (Titova 1973: 110): “Kazakhstan, Trans-Ili Alatau… Moreover, *E.retusidens* were studied from the Dzhungarian Alatau, the Greater Caucasus, Crimea, Moldova, Rostov, Voronezh and Voroshilovograd oblasts”.

### ﻿36. Escaryus sachalinus

Taxon classificationAnimaliaGeophilomorphaSchendylidae

Takakuwa, 1935

753ECBF4-3359-5B8C-BFA8-7FE922A7E429

Escaryus sachalinus Takakuwa 1935: 48.
Escaryus sachalinus – Titova 1969: 165; 1972: 135; 1973: 94, 118; Shinohara 1972: 66; Kurcheva 1977: 45.

#### Type localities.

Russia: Sakhalin oblast: “Sachalin” = Sakhalin Isl. Japan: Hokkaido Isl.: “Sapporo” (both Takakuwa 1935).

#### Type series.

***Syntypes***: 3 specimens. Depository unknown.

#### Diagnosis.

An *Escaryus* species with body length reaching ≥ 3 cm; clypeus without plagulae; labral arc relatively deep, with denticles short and obtuse; first maxillae with lappets; all forcipular articles with small denticles, except tarsungulum; 35–39 leg-bearing segments; metasternite of the ultimate leg-bearing segment rectangular, ~ 1.5× as long as wide; numerous coxal pores on ventral side of coxopleuron; anal pores present.

#### Distribution.

Far East: Sakhalin oblast (Sakhalin Isl.) (Titova 1973; Kurcheva 1977). Outside Asian Russia: Japan (Takakuwa 1935; Shinohara 1972) and northern China (Shinohara 1972).

### ﻿37. Escaryus sibiricus

Taxon classificationAnimaliaGeophilomorphaSchendylidae

Cook, 1899

B9897045-E641-5D7E-9857-F8FC4CDA1A0A

Escaryus sibiricus Cook 1899: 304.
Escaryus sibiricus – Attems 1904: 122; 1927: 301; 1929: 95; Titova 1972: 135; 1973: 94, 113; Ganin 1997: 141; Thofern et al. 2021: 30.

#### Type locality.

Russia: Maritime krai: “Vladivostock” (Cook 1899) = Vladivostok city, 43°7'N, 131°54'E.

#### Type series.

***Syntypes***: 12 specimens, both sexes, including 10 specimens deposited in ZMH (Thofern et al. 2021) and 2 specimens deposited in NHMW (Ilie et al. 2009).

#### Diagnosis.

An *Escaryus* species with body length reaching 6.5 cm; labral arc relatively shallow, with middle denticles obtuse and lateral ones long and pointed; first maxillae with two pairs of lappets; forcipular trochanteroprefemur with a small distal bulge, other forcipular articles without denticles; 49–51 leg-bearing segments; metasternite of the ultimate leg-bearing segment rectangular, ~ 2× as long as wide; coxal pores of different size, on both ventral and lateral sides, including a pair of much larger ventral pores on each coxopleuron; anal pores absent.

#### Distribution.

Far East: Maritime krai (Cook 1899; Attems 1904) and Amur oblast (Ganin 1997). Outside Asian Russia: no records.

#### Remarks.

Titova (1969) reported this species from Western Siberia (Kemerovo oblast) but later (Titova 1973) she regarded the same record as *E.koreanus*, even though she also suggested that the latter species can be a junior synonym of *E.sibiricus*. The anal pores were indicated as absent by Cook (1899) and Attems (1904) but illustrated as present in specimens identified as *E.sibiricus* by Thofern et al. (2021), and they are known to be present in *E.koreanus*.

### ﻿38. Escaryus vitimicus

Taxon classificationAnimaliaGeophilomorphaSchendylidae

Titova, 1973 *

92A0792F-BCB6-55A7-B2AB-1CF390718C38

Escaryus vitimicus Titova 1973: 103.
Escaryus vitimicus – Alekseeva 1974: 8.

#### Type locality.

Russia: Republic of Buryatia: “r. Vitim, Aniboli” (Titova 1973) = ? Anibud river, inflow of Vitim River, ca 53°39'N, 113°53'E.

#### Type series.

***Holotype***: female. Paratypes: 3 specimens, including 1 male and 2 females. Deposited in ZMMU (S. Golovatch and A. Schileyko, pers. comm., 13.II.2023 and XII.2023).

#### Diagnosis.

An *Escaryus* species with body length reaching ≥ 2.7 cm; clypeus with large plagulae; labral arc relatively shallow, with denticles long and obtuse; first maxillae with one pair of lappets; all forcipular articles with small bulges; 37 leg-bearing segments; metasternites with relatively sparse setae; metasternite of the ultimate leg-bearing segment rectangular, ~ 1.5× as long as wide; coxal pores on both ventral and lateral sides of coxopleura, the largest pores near the metasternite; anal pores present.

#### Distribution.

Eastern Siberia: Republic of Buryatia (Titova 1973; Alekseeva 1974). Outside Asian Russia: no records.

##### ﻿Other records of uncertain species

During ecological studies (Ghilarov and Perel 1973; Molodova 1973; Kurcheva 1977; Markelov and Mineeva 1981; Ganin 1997) some specimens of Geophilomorpha were not identified at the species level. These records are listed below.

*Arctogeophilus* sp. – Ghilarov and Perel 1973: 46 (Maritime krai); Markelov and Mineeva 1981: 130 (Maritime krai).

*Geophilus* sp. – Molodova 1973: 67 (Sakhalin oblast: Sakhalin Isl.); Ganin 1997: 105, 109, 112, 114, 128, 141 (Amur oblast and Khabarovsk krai); Markelov and Mineeva 1981: 130 (Maritime krai).

*Pachymerium* sp. – Kurcheva 1977: 46 (Khabarovsk and Maritime krais).

*Scolioplanes* sp. – Ghilarov and Perel 1973: 46 (Maritime krai); Alekseeva 1974: 8 (Republic of Buryatia); Markelov and Mineeva 1981: 130 (Maritime krai).

*Strigamia* sp. – Markelov and Mineeva 1981: 130 (Maritime krai).

*Prolamnonyx* sp. – Kurcheva 1977: 46 (Maritime krai and Sakhalin oblast: Kuril Islands).

*Escaryus* sp. – Markelov and Mineeva 1981: 129 (Maritime krai).

## ﻿Discussion

### ﻿History of studies

The first record of Geophilomorpha from Asian Russia was published by Gerstfeldt (1859), who recorded *Arthronomaluslongicornis* Leach, 1815 (=*Geophilusflavus* (De Geer, 1778)) from the Zabaykalsky krai.

Other records of Geophilidae were provided by Meinert (1870), who described *Geophiluspilosus* from Sakhalin Isl., and by Stuxberg (1876a, 1876b), who recorded the latter species in the Krasnoyarsk krai and also described *G.sibiricus* from this region. Around the same time Sseliwanoff (1881) described *G.orientalis* from the Khabarovsk krai, and recorded *G.pilosus* from the Krasnoyarsk krai and Irkutsk oblast, and *G.proximus* C.L. Koch, 1847 from Western Siberia. Attems (1909) described *Arctogeophilusglacialis* from three localities (two of them in the Chukotka Peninsula). Later, Verhoeff (1934) and Takakuwa (1937c) described *A.sachalinus*, *Geophilusbipartitus* and *G.rhomboideus* from Sakhalin Isl. Numerous faunistic and ecological studies (Byzova and Chadaeva 1965; Ghilarov and Perel 1973; Molodova 1973; Kurcheva 1977; Markelov and Mineeva 1981; Zalesskaja et al. 1982; Poryadina 1991; Striganova and Poryadina 2005; Ganin 1997, 2006; Vorobiova 1999; Vorobiova et al. 2002; Rybalov 2002; Farzalieva 2008; Sergeeva 2013; Bukhkalo and Sergeeva 2012; Bukhkalo et al. 2014; Nefediev et al. 2017a, 2017b, 2017c, 2018, 2021; Nefediev 2019) provided additional data for seven species of geophilids from Asian Russia: *Arctogeophilusmacrocephalus* Folkmanová & Dobroruka, 1960, *Geophilusbipartitus*, *G.flavus*, *G.proximus*, *G.rhomboideus*, *G.sounkyoensis* Takakuwa, 1937, and *Pachymeriumferrugineum* (C.L. Koch, 1835).

The first record of *Strigamia* Gray, 1843 from Asian Russia was published by Meinert (1870), who described *Scolioplanessacolinensis* from Sakhalin Isl. Later, Sseliwanoff (1881) described *S.sibiricus* from the Zabaykalsky krai and *S.sulcatus* from the Khabarovsk krai. A number of papers (Ghilarov and Perel 1973; Molodova 1973; Kurcheva 1977; Ganin 1997; Farzalieva 2008; Nefediev et al. 2017c, 2018; Nefediev 2019) provided other distribution data for seven *Strigamia* species from Asian Russia: *S.acuminata*, *S.alokosternum*, *S.hirsutipes*, *S.pusilla*, *S.sacolinensis*, *S.sulcata*, and *S.transsilvanica*.

The first record of the family Mecistocephalidae was published by Titova (1969), who reported a specimen from the Maritime krai, first as *Prolamnonyxholstii* (Pocock, 1895) and later (Titova 1975) as a new species, *Prolamnonyxmamaevi*. She also described *Krateraspisstriganovae* from the Maritime krai and recorded *Prolamnonyxdentatus* from Far East. Markelov and Mineeva (1981) and Ganin (1997, 2011) published additional data for the species mentioned by Titova; Nefediev (2019) recorded an anthropochore introduction, *Tygarrupjavanicus* Attems, 1929, in the Altai krai.

The first species of *Escaryus* Cook & Collins, 1891 (Schendylidae), *E.sibiricus*, was described by Cook (1899) from Vladivostok (Maritime krai). Later, Attems (1904) redescribed this species based on the type material, and Takakuwa (1935) described another species, *E.sachalinus*, from Sakhalin Isl. Eight other species of *Escaryus* were described by Titova (1973): *E.molodovae* and *E.hirsutus* from Sakhalin Isl.; *E.dentatus*, *E.krivolutskiji*, *E.perelae*, and *E.polygonatus* from the Maritime krai; *E.vitimicus* from the Republic of Buryatia; *E.chadaevae* from the Kemerovo oblast. Some ecological and faunistic papers (Titova 1972, 1973; Ghilarov and Perel 1973; Molodova 1973; Alekseeva 1974; Kurcheva 1977; Markelov and Mineeva 1981; Rybalov 2002; Ganin 1997, 2006; Vorobiova et al. 2002; Dyachkov 2017; Nefediev et al. 2017a, 2017c, 2018; Nefediev 2019) provided new data of *Escaryus* species, including four other species, namely *E.chichibuensis* Shinohara, 1955, *E.japonicus* Attems, 1927, *E.koreanus* Takakuwa, 1937, and *E.retusidens* Attems, 1904.

### ﻿State of knowledge

The published records of Geophilomorpha from Asian Russia refer to 38 nominal species, arranged in eight genera (Table 1). However, the taxonomic validity of ≥ 19 species is uncertain, including 14 species that are known from Asian Russia only (Table 1). The taxonomic status of most of these species has never been revised since they were originally described. Of all the species reported from Asian Russia, only nine are also known from European Russia, where, a total of 41 species from 17 genera have been reported so far (Volkova 2016; Zuev and Evsyukov 2016; Dyachkov and Bonato 2022).

The records of *Tygarrupjavanicus* and *Geophilusflavus* from hothouses in Western Siberia by Nefediev et al. (2017a; Nefediev 2019) seems to be due to anthropochore introduction. However, the occurrence of *Geophilusflavus* outside hothouses in Asian Russia requires confirmation. Gerstfeldt (1859) identified a sole specimen from Eastern Siberia (Zabaykalsky krai) as *Arthronomaluslongicornis* (= *Geophilusflavus*), but Sseliwanoff (1884: 90) questioned this identification. Moreover, the nominal species *Schizotaeniaornata* Folkmanová & Dobroruka, 1960 was mentioned by Zalesskaja et al. (1982) from Western Siberia, however without providing information on specimens or published sources. This nominal species was considered a junior synonym of *Geophilusproximus* by Zalesskaja et al. (1982), but was later synonymized under *G.flavus* by Bonato and Minelli (2014). Nefediev et al. (2017a) suggested that some previous records of *G.flavus* from the former USSR may be reported under the name *G.proximus*.

Other species require confirmation from Asian Russia. The records of *Strigamiaacuminata* from Far East and *S.transsilvanica* from Western Siberia and Far East are dubious because of possible confusion with other species (Bonato et al. 2012, 2023). Moreover, Titova (1972, 1973) indicated the presence of *Escaryusretusidens* from the Russian Far East, but she did not mention material from that region when she listed the studied specimens. Nefediev et al. (2017a, 2017c, 2018) indicated this species from the Far East with reference to Titova (1972, 1973). It is worth noting that *E.retusidens* has never been recorded during ecological studies in Eastern Siberia (Alekseeva 1974; Vorobiova 1999; Vorobiova et al. 2002; Rybalov 2002) or the Far East (Kurcheva 1977; Ganin 1997, 2006, 2011).

Our synthesis of all published information on Geophilomorpha from Asian Russia shows that the knowledge of this fauna is very far from being satisfactory. We hope that this work may provide a background reference and will prompt further investigations.

## Supplementary Material

XML Treatment for Arctogeophilus

XML Treatment for Arctogeophilus glacialis

XML Treatment for Arctogeophilus macrocephalus

XML Treatment for Arctogeophilus sachalinus

XML Treatment for Geophilus

XML Treatment for Geophilus bipartitus

XML Treatment for Geophilus flavus

XML Treatment for Geophilus orientalis

XML Treatment for Geophilus proximus

XML Treatment for Geophilus rhomboideus

XML Treatment for Geophilus sibiricus

XML Treatment for Geophilus sounkyoensis

XML Treatment for Pachymerium

XML Treatment for Pachymerium ferrugineum

XML Treatment for Pachymerium pilosum

XML Treatment for Strigamia

XML Treatment for Strigamia cf. acuminata

XML Treatment for Strigamia alokosternum

XML Treatment for Strigamia hirsutipes

XML Treatment for Strigamia pusilla

XML Treatment for Strigamia sacolinensis

XML Treatment for Strigamia sibirica

XML Treatment for Strigamia sulcata

XML Treatment for Strigamia cf. transsilvanica

XML Treatment for Agnostrup

XML Treatment for Agnostrup striganovae

XML Treatment for Arrup

XML Treatment for Arrup dentatus

XML Treatment for Arrup mamaevi

XML Treatment for Tygarrup

XML Treatment for Tygarrup javanicus

XML Treatment for Escaryus

XML Treatment for Escaryus chadaevae

XML Treatment for Escaryus chichibuensis

XML Treatment for Escaryus dentatus

XML Treatment for Escaryus hirsutus

XML Treatment for Escaryus japonicus

XML Treatment for Escaryus koreanus

XML Treatment for Escaryus krivolutskiji

XML Treatment for Escaryus molodovae

XML Treatment for Escaryus perelae

XML Treatment for Escaryus polygonatus

XML Treatment for Escaryus retusidens

XML Treatment for Escaryus sachalinus

XML Treatment for Escaryus sibiricus

XML Treatment for Escaryus vitimicus

## ﻿Additional information

### Author ORCIDs

Yurii V. Dyachkov https://orcid.org/0000-0001-9256-9306

Lucio Bonato https://orcid.org/0000-0002-8312-7570

### Data availability

All of the data that support the findings of this study are available in the main text.

## References

[B1] Alekseeva EE (1974) Soil mesofauna of steppes and forests in western Transbaikalia. Autoreferate of PhD Thesis. Moscow, 20 pp. [In Russian]

[B2] Attems C (1903a) Myriopoden. Fauna Arctica III(1): 35–54.

[B3] Attems C (1903b) Synopsis der Geophiliden. Zoologischen Jahrbüchern, Abtheilung für Systematik. Geographie und Biologie der Thiere 18(2): 155–302.

[B4] Attems C (1904) Central- und hoch-asiatische Myriopoden. Gesammelt im Jahre 1900 von Dr. von Almassy und Dr. von Stummer. Zoologische Jahrbucher. Abteilung fur Systematik, Ökologie und Geographie der Tiere 20: 113–130. 10.5962/bhl.part.18578

[B5] Attems C (1909) Die Myriopoden der Vega-Expedition. Arkiv för Zoologi 5(3): 1–84. 10.5962/bhl.part.3495

[B6] Attems C (1927) Neue Chilopoden. Zoologischer Anzeiger 72: 291–305.

[B7] Attems C (1929) Myriapoda. 1. Geophilomorpha. Das Tierreich 52: 1–388. 10.1515/9783111430638

[B8] Barber AD (2009) Centipedes. Synopses of the British Fauna, 58, The Linnean Society of London, 228 pp.

[B9] Berman DI Leirikh AN (2019) Overwintering and cold hardiness of the invertebrates in the Northeast Asia. KMK Scientific Press, 314 pp.

[B10] Bonato L (2011) Chilopoda. Taxonomic overview. Order Geophilomorpha In: Minelli A (Ed.) Treatise on zoology – anatomy, taxonomy, biology.The Myriapoda. Volume 1. Brill, Leiden-Boston, 407–443.

[B11] Bonato L Minelli A (2014) ChilopodaGeophilomorpha of Europe: a revised list of species, with taxonomic and nomenclatorial notes. Zootaxa 3770: 136. 10.11646/zootaxa.3770.1.1 24871280

[B12] Bonato L Foddai D Minelli A Shelley R (2004) The centipede order Geophilomorpha in the Hawaiian Islands (Chilopoda). Bishop Museum Occasional Papers 78: 13–32.

[B13] Bonato L Minelli A Spuņģis V (2005) Geophilomorph centipedes of Latvia (Chilopoda, Geophilomorpha). Latvijas Entomologs 42(1): 5–17.

[B14] Bonato L Dányi L Socci AA Minelli A (2012) Species diversity of *Strigamia* Gray, 1843 (Chilopoda: Linotaeniidae): a preliminary synthesis. Zootaxa 3593(1): 1–39. 10.11646/zootaxa.3593.1.1

[B15] Bonato L Drago L Murienne J (2014) Phylogeny of Geophilomorpha (Chilopoda) inferred from new morphological and molecular evidence. Cladistics 30(5): 485–507. 10.1111/cla.12060 34794246

[B16] Bonato L Chagas Jr A Edgecombe GD Lewis JGE Minelli A Pereira LA Shelley RM Stoev P Zapparoli M (2016) ChiloBase 2.0 – A World Catalogue of Centipedes (Chilopoda). https://chilobase.biologia.unipd.it

[B17] Bonato L Bortolin F De Zen G Decker P Lindner EN Orlando M Spelda J Voigtländer K Wesener T (2023) Towards elucidating species diversity of European inland *Strigamia* (Chilopoda: Geophilomorpha): a first reassessment integrating multiple lines of evidence. Zoological Journal of the Linnean Society 199(4): 945–966. 10.1093/zoolinnean/zlad070

[B18] Bragina TM Dyachkov YuV Farzalieva GS (2020) New data on the centipede fauna (Myriapoda: Chilopoda) of Kostanay region, Kazakhstan. Far Eastern Entomologist = Dal’nevostochnyi Entomolog 406: 27–32. 10.25221/fee.406.4

[B19] Brolemann HW (1930) Éléments d’une faune des Myriapodes de France. Chilopodes. Faune de France 25: 1–405.

[B20] Bukhkalo SP Sergeeva EV (2012) Interannual dynamics of the composition and structure of soil invertebrate communities in the root terrace of the Irtysh. Belgorod State University Scientific Bulletin. Natural sciences 15(134/20): 59–64. [In Russian, with English summary]

[B21] Bukhkalo SP Galitch DE Sergeeva EV Vazhenina NV (2014) Synopsis of the invertebrate fauna of the southern taiga in western Siberia (the basin of the Lower Irtysh). KMK Press, Moscow, 189 pp. [In Russian]

[B22] Byzova YuB Chadaeva ZV (1965) A comparative characteristic of the soil fauna of various associations in the *Abiessibirica* forest (Kemerovo oblast). Zoologicheskii Zhurnal 44(3): 331–339. [In Russian, with English summary]

[B23] Chamberlin RV (1919) The Chilopoda collected by the Canadian Arctic Expedition, 1913–18. In: Report of the Canadian Arctic Expedition, 1913–1918. Volume III: Insects. Part H: Spiders, mites, and myriapods. J. de Labroquerie Taché, Ottawa, 15–22.

[B24] Chamberlin RV (1946) On the chilopods of Alaska. Annals of the Entomological Society of America 39(2): 177–189. 10.1093/aesa/39.2.177

[B25] Cook OF (1896) An arrangement of the Geophilidae, a family of Chilopoda. Proceedings of the United States National Museum 18(1039): 63–75. 10.5479/si.00963801.18-1039.63

[B26] Cook OF (1899) The Geophiloidea of Florida Keys. Proceedings of the Entomological Society of Washington 4: 303–312.

[B27] Crabill RE (1964) A revised interpretation of the primitive centipede genus *Arrup*, with redescription of its type-species and list of known species. Proceedings of the Biological Society of Washington 77: 161–170.

[B28] Daday E (1889) Myriopoda extranea Musaei Nationalis Hungarici. Természetrajzi Füzetek 12: 114–156.

[B29] Damasiewicz A Leśniewska M (2020) *Tygarrupjavanicus* (Chilopoda, Geophilomorpha) – an exotic species that has reached Polasandrt. Polskie Pismo Entomologiczne 89(1): 52–58. 10.5604/01.3001.0014.0300

[B30] Dobroruka LJ (1955) Poznámky k československým zástupcům rodu *Scolioplanes* (Chilopoda). Sborník Krajského Vlastivedného Musea v Olomouci 3A: 201–204.

[B31] Dyachkov YuV (2017) The first data on centipede (Chilopoda: Geophilomorpha; Lithobiomorpha) fauna of the Katunskiy Biosphere State Nature Reserve, Altai Mts. Ukrainian Journal of Ecology 7(4): 453–456. 10.15421/2017_141 [in Russian, with English summary]

[B32] Dyachkov YuV (2018a) New data on the distribution of *Pachymeriumferrugineum* (C.L. Koch, 1835) (Chilopoda: Geophilomorpha: Geophilidae) in Central Asia. Ukrainian Journal of Ecology 8(4): 252–254. [In Russian, with English summary]

[B33] Dyachkov YuV (2018b) Linotaeniidae Cook, 1899 (Chilopoda: Geophilomorpha), a new family to the fauna of Kazakhstan. Ukrainian Journal of Ecology 8(4): 255–257.

[B34] Dyachkov YuV (2020) New data on the centipede (Chilopoda) fauna from Tajikistan. Ecologica Montenegrina 36: 78–86. 10.37828/em.2020.36.6

[B35] Dyachkov YuV (2022) On new records of Geophilomorpha (Chilopoda) from Middle Asia. Ecologica Montenegrina 60: 70–79. 10.37828/em.2022.60.11

[B36] Dyachkov YuV (2023) To the fauna of Geophilomorpha (Chilopoda) of Uzbekistan and Turkmenistan. Acta Biologica Sibirica 9: 1073–1082. 10.5281/zenodo.10239340

[B37] Dyachkov YuV Bonato L (2022) Morphology and distribution of the Middle Asian centipede genus *Krateraspis* Lignau, 1929 (Chilopoda, Geophilomorpha, Mecistocephalidae). ZooKeys 1095: 143–164. 10.3897/zookeys.1095.80806 35836682 PMC9023436

[B38] Dyachkov YuV Farzalieva GS (2023) An annotated checklist of Chilopoda from Mongolia. Ecologica Montenegrina 64: 221–241. 10.37828/em.2023.64.7

[B39] Dyachkov YuV Nedoev KhKh (2021) A contribution to the centipede (Chilopoda: Geophilomorpha, Scolopendromorpha) fauna of Uzbekistan and Turkmenistan. Ecologica Montenegrina 41: 41–50. 10.37828/em.2021.41.6

[B40] Dyachkov YuV Tuf IH (2018) New data on the genus *Escaryus* Cook et Collins, 1891 (Chilopoda: Geophilomorpha: Schendylidae) from Kazakhstan. Arthropoda Selecta 26(4): 293–299. 10.15298/arthsel.27.4.04

[B41] Dyachkov YuV Tuf IH (2019) New data on the family Geophilidae Leach, 1815 (Chilopoda: Geophilomorpha) from Kazakhstan. Far Eastern Entomologist = Dal’nevostochnyi Entomolog 391: 24–28. 10.25221/fee.391.2

[B42] Dyachkov YuV Zuev RV (2023) Myriapoda (Chilopoda, Diplopoda) of the South Ossetia. Acta Biologica Sibirica 9: 157–165. 10.5281/zenodo.7825736

[B43] Dyachkov YuV Zuev RV Gichikhanova UA (2022) Centipedes (Chilopoda) from the Dagestan, Northern Caucasus, Russia. Ecologica Montenegrina 52: 68–89. 10.37828/em.2022.52.10

[B44] Dyachkov Yu V Ali Al-Yacoub GA Al-Khazali AM (2023) A preliminary annotated checklist of Chilopoda from Iraq. Ecologica Montenegrina 63: 59–78. 10.37828/em.2023.63.6

[B45] Farzalieva GSh (2008) The fauna and chorology of Myriapoda from the Urals and Cisuralia. PhD Thesis. Perm State University, Perm, 189 pp. [In Russian]

[B46] Foddai D Bonato L Pereira LA Minelli A (2003) Phylogeny and systematics of the Arrupinae (ChilopodaGeophilomorphaMecistocephalidae) with the description of a new dwarfed species. Journal of Natural History 37(10): 1247–1267. 10.1080/00222930210121672

[B47] Folkmanová B Dobroruka LJ (1960) Contribution to knowledge of centipedes (Chilopoda) of USSR. Zoologicheskii Zhurnal 39(12): 1811–1818. [In Russian with German summary]

[B48] Ganin GN (1997) Soil Invertebrates of the Ussuri Taiga. Dalnauka, Vladivostok-Khabarovsk, 160 pp. [In Russian, with English summary]

[B49] Ganin GN (2006) Some rules of soil invertebrate community organization (by the Example of Amur Basin Mesofauna). Biology Bulletin of the Russian Academy of Sciences 33(5): 498–507. 10.1134/S1062359006050128 [In Russian, with English summary] 17086972

[B50] Ganin GN (2011) Structural and functional organization of mezopedobiont communities of the southern Russian Far East. Dalnauka, Vladivostok, 380 pp. [In Russian, with English summary]

[B51] Gerstfeldt G (1859) Ueber einige zum Theil neue Arten Platoden, Anneliden, Myriapoden und Crustaceen Sibiriens, namentlich seines ¨ostlichen Theiles und des Amur-Gebiets. Mémoires de l’Académie impériale des sciences de St. Pétersbourg 8: 259–296.

[B52] Ghilarov MS Perel TS (1973) Complexes of soil invertebrates of coniferous-deciduous forests of Far East as indicator of soil type. In: Ghilarov MS (Ed.) Ekologiya pochvennykh bespozvonochnykh.Nauka Publisher, Moscow, 40–59. [In Russian]

[B53] Gilgado JD Cabanillas D Bobbitt I (2022) Millipedes and centipedes (Myriapoda: Diplopoda, Chilopoda) in Swiss heated greenhouses, with seven species new for Switzerland. Revue Suisse de Zoologie 129(1): 85–101. 10.35929/RSZ.0063

[B54] Gvozdetskiy NA Mikhailov NI (1978) Physical geography of USSR. Asian part. Misl Publisher, Moscow, 512 pp. [In Russian]

[B55] Ilie V Schiller E Stagl V (2009) Type specimens of the Geophilomorpha (Chilopoda) in the Natural History Museum in Vienna. Kataloge der wissenschaftlichen Sammlungen des Naturhistorischen Museum in Wien. Verlag des Naturhistorischen Museum in Vienna 22(4): 1–75.

[B56] Iorio E (2005) *Strigamiatranssilvanica* (Verhoeff, 1928), espéce nouvelle pour la faune de France (Chilopoda, Geophilomorpha, Linotaeniidae). Bulletin de la Société linnéenne de Bordeaux 33: 195–198.

[B57] Jonishi T Nakano T (2022) Correct authorships, synonymies, and remarks on the type series of fourteen names of Centipedes introduced by Yoshioki Takakuwa in 1934 and *Mecistocephalustakakuwai* (Chilopoda: Geophilomorpha and Scolopendromorpha). Species Diversity: An International Journal for Taxonomy, Systematics, Speciation, Biogeography, and Life History Research of Animals 27(1): 71–81. 10.12782/specdiv.27.71

[B58] Kazantsev SV (2022) To the translation of names of Russian administrative divisions into English. Evraziatskii Entomologicheskii Zhurnal 31(4): 436–440. 10.15298/rusentj.31.4.16

[B59] Kurcheva GF (1977) Soil invertebrates of Soviet Far East. Nauka Publisher, Moscow, 132 pp. [In Russian]

[B60] Langor DW Langor SD (2022) The biota of Canada: checklist of the centipedes of Canada (Myriapoda: Chilopoda). Canadian Entomologist 154(1, e8): 1–12. 10.4039/tce.2021.58

[B61] Leach WE (1815) A tabular view of the external characters of four classes of animals, which Linné arranged under Insecta; with the distribution of the genera composing three of these classes into orders, and descriptions of several new genera and species. Transactions of the Linnean Society of London 11(2): 306–400. 10.1111/j.1096-3642.1813.tb00065.x

[B62] Loksa I (1962) Zwei neuen Chilopoden-Formen aus der Umgebung von Rybinsk. Zoologicheskij Zhurnal 41: 854–858.

[B63] Markelov AV Mineeva NY (1981) Features of the formation of complexes of geophilomorph centipedes in the subzone of pine-broad-leaved forests (Sikhote-Alin). Problemy pochvennoi zoologii. Materials of the VII All-Union Conference. Kiev, 129–130. [In Russian]

[B64] Meinert F (1870) Myriapoda Musaei Hauniensis: Bidrag til Myriapodernes morphologi og systematik. I. Geophili. Naturhistorisk Tidsskrift 7(3): 1–128.

[B65] Molodova LP (1973) Fauna of soil invertebrates of southern Sakhalin. In: Ghilarov MS (Ed.) Ekologiya pochvennykh bespozvonochnykh.Nauka Publisher, Moscow, 60–74. [In Russian]

[B66] Murakami Y (1993) Chilopoda. In: List of animals and plants in Japan. Invertebrates 1. Environment Agency Japan, Nature and Environment Research Center, Tokyo, 103–105.

[B67] Muralewicz WS (1926) Übersicht über die Chilopodenfauna des Kaukasus. II. Mitteilung. Zoologischer Anzeiger 69: 27–44.

[B68] Nefediev PS (2019) New records of geophilomorph centipedes (Chilopoda: Geophilomorpha) from natural and anthropogenic habitats of Siberia. Far Eastern Entomologist = Dal’nevostochnyi Entomolog 380: 23–28. 10.25221/fee.380.4

[B69] Nefediev PS Tuf IH Farzalieva GS (2017a) Centipedes from urban areas in southwestern Siberia, Russia (Chilopoda). Part 2. Geophilomorpha. Arthropoda Selecta 26(1): 8–14. 10.15298/arthsel.26.1.02

[B70] Nefediev PS Knyazev SY Farzalieva GS Tuf IH (2017b) A contribution to the myriapod fauna of the Omsk Area, Siberia, Russia (Myriapoda: Diplopoda, Chilopoda). Arthropoda Selecta 26(2): 113–118. 10.15298/arthsel.26.2.03

[B71] Nefediev PS Farzalieva GS Tuf IH (2017c) A preliminary review of the centipede fauna of the Altai State Nature Biosphere Reserve, southwestern Siberia, Russia (Chilopoda: Lithobiomorpha, Geophilomorpha). Arthropoda Selecta 26(3): 217–224. 10.15298/arthsel.26.3.02

[B72] Nefediev PS Farzalieva GS Tuf IH Nedoev KK Niyazov ST (2018) Millipede and centipede assemblages on the northern and southern slopes of the lowland Altais, southwestern Siberia, Russia (Diplopoda, Chilopoda). ZooKeys 741: 219–254. 10.3897/zookeys.741.21936 PMC5904556 29706778

[B73] Nefediev PS Nefedieva JS Farzalieva GS (2021) New data on the myriapod fauna (Myriapoda: Chilopoda, Diplopoda) of the Republic of Khakassia, central Siberia, Russia. Zoologia Bespozvonocnyh 18(1): 36–46. 10.15298/invertzool.18.1.04

[B74] Nordenskiöld AE (1882) The voyage of the Vega round Asia and Europe. Macmillan and Company, New York, 756 pp.

[B75] Poloczek A Pfeiffer M Schneider R Müchlenberg M (2016) The Chilopoda (Myriapoda) of the Khentey-Mountain Range, Northern Mongolia. Communities of different forest-types under a varying fire regime. European Journal of Soil Biology 74: 114–120. 10.1016/j.ejsobi.2016.04.004

[B76] Poryadina NM (1991) Mesofauna of forest soils in the Western Siberian Plain. Tomsk State University, Tomsk, 21 pp. [In Russian]

[B77] Reip HS Voigtländer K (2009) Diplopoda and Chilopoda of Thuringia. Soil Organisms 81(3): 635–645.

[B78] Rybalov LB (2002) Zonal and landscape changes in soil invertebrate populations in a near-Yenisei River region of middle Siberia and the role of temperature adaptations in the meridional (zonal) distribution of invertebrates. Russian Entomological Journal 11(1): 77–86. [In Russian, with English summary]

[B79] Sergeeva EV (2013) Biotopic distribution and the numbers of centipedes (Chilopoda) in the Irtysh Valley of western Siberia, Russia. Evraziatskii Entomologicheskii Zhurnal 12(6): 529–533. [In Russian, with English summary]

[B80] Sergeeva EV (2014) Species diversity of soil invertebrate communities in the Irtysh root terrace. Belgorod State University Scientific Bulletin. Natural Sciences 17(188/28): 70–75. [In Russian, with English summary]

[B81] Shinohara K (1955) On the chilopods at the Chichibu Mountains. Bulletin of the Chichibu Museum of Natural History 6: 54–62.

[B82] Shinohara K (1972) Some Chilopods of the Hidaka Mountain Range in Hokkaido, Northern Japan. Memoirs of the National Science Museum, Tokyo 5: 65–73.

[B83] Sseliwanoff AV (1881) Geophilidae from the Museum of Imperial Academy of Sciences. Zapiski Imperatorskoi Akademii Nauk 40: 1–27. [In Russian]

[B84] Sseliwanoff AV (1884) Materials towards the study of Russian myriapods. Trudy Russkago Entomologicheskago Obshchestva 18(1–2): 69–121. [In Russian]

[B85] Stoev P (2007) Fauna and Zoogeography of Myriapoda in Bulgaria. In: Fet V Popov A (Eds) Biogeography and ecology of Bulgaria.Springer, 379–404. 10.1007/978-1-4020-5781-6_11

[B86] Striganova BR Poryadina NM (2005) Soil animal population in boreal forests of the West Siberian Plain. KMK Press, Moscow, 234 pp. [In Russian]

[B87] Stuxberg A (1871) Bidrag till Skandinaviens Myriopodologi. II. Sveriges Chilopoder. Öfversigt af Kongliga Vetenskaps-Akademiens Förhandlingar 28: 493–512.

[B88] Stuxberg A (1876a) Myriopoder från Sibirien och Waigatsch ön samlade under Nordenskiöldska expeditionen 1875. Öfversigt af Kongl. Vetenskaps-akademiens Förhandlingar 33(2): 11–38.

[B89] Stuxberg A (1876b) On the Myriopoda, from Siberia and Waigatsch Island, collected during the expedition of Prof. Nordenskiöld, 1875. Annals & Magazine of Natural History 4(17): 306–318. 10.1080/00222937608681955

[B90] Takakuwa Y (1933) Myriapods of the Northern Kurile Islands. Nihon Seibutsu Chiri Gakkai Kaiho 4(2): 133–137.

[B91] Takakuwa Y (1934a) Japanese Mecistocephalidae (I). Shokubutsu oyobi Dobutsu 2: 706–712. [In Japanese]

[B92] Takakuwa Y (1934b) Neue Japanische Mecistocephalidae. Annotationes Zoologicae Japonenses 14: 355–363.

[B93] Takakuwa Y (1934c) Japanese Mecistocephalidae (II). Botany and Zoology 2(5): 878–884. [In Japanese]

[B94] Takakuwa Y (1935) Über Japanische *Escaryus*-Arten. Transactions of the Sapporo Natural History Society 14: 46–50.

[B95] Takakuwa Y (1937a) Eine neue *Escaryus*-Art aus Korea. Zoological Magazine 49: 297–299.

[B96] Takakuwa Y (1937b) The *Geophilus*-species of Japan. Zoological Magazine 49: 282–286.

[B97] Takakuwa Y (1937c) *Geophilus*-Arten aus Japan. Transactions of the Sapporo Natural History Society XV(2): 76–81.

[B98] Takakuwa Y (1938) Über die japanischen Scolioplanes (Chilopoda)-Arten. Zoological Magazine 50: 235–245.

[B99] Takakuwa Y (1940) Geophilomorpha. In: Okada Y et al. (Eds) Fauna Nipponica, 9, fasc. 8(1), Sanseido, Tokyo, 1–156.

[B100] Takakuwa Y Takashima H (1949) Myriapods collected in Shansi, North China. Acta Arachnologica 11(1–2): 51–69. 10.2476/asjaa.11.51 [In Japanese, with English summary]

[B101] Thofern D Dupérré N Harms D (2021) An annotated type catalogue of the centipedes (Myriapoda: Chilopoda) held in the Zoological Museum Hamburg. Zootaxa 4977(1): 1–103. 10.11646/zootaxa.4977.1.1 34187023

[B102] Titova LP (1969) Geophilids of the USSR fauna and news in the distribution of the fam. Mecistocephalidae. In: Aleinikova MM (Ed.) Problems of soil zoology.Materials of the 3^th^ All-Union Conference, Kazan. Nauka Publisher, Moscow, 165–166. [In Russian]

[B103] Titova LP (1972) Pattern of the distribution of the genus *Escaryus* (Chilopoda) in the USSR. In: Ghilarov MS (Ed.) Problemy pochvennoi zoologii.Materials of the 4^th^ All-Union Conference. Baku, 1972. Nauka Publisher, Moscow, 135–136. [In Russian]

[B104] Titova LP (1973) New species of the genus *Escaryus* Cook et Collins (Schendylidae, Chilopoda). In: Ghilarov MS (Ed.) Ekologiya pochvennykh bespozvonochnykh.Nauka Publisher, Moscow, 94–119. [In Russian]

[B105] Titova LP (1975) Geophilids of the family Mecistocephalidae in the USSR fauna (Chilopoda). Zoologicheskii Zhurnal 54(1): 39–48. [In Russian]

[B106] Tuf IH Kupka J (2015) First record of *Strigamiapusilla* from the Czech Republic (Chilopoda: Geophilomorpha). Acta Carpathica Occidentalis 6(1): 108–110. 10.62317/aco.2015.009

[B107] Tuf IH Tajovsky K (2016) An annotated checklist of the centipedes (Chilopoda) recorded in the Czech Republic. Acta Societatis Zoologicae Bohemoslovacae 80: 45–50.

[B108] Tuf IH Mock A Dvořák L (2018) An exotic species spreads through Europe: *Tygarrupjavanicus* (Chilopoda: Geophilomorpha: Mecistocephalidae) is reported from the Slovakia and the Czech Republic. Journal of Asia-Pacific Entomology 21(2): 560–562. 10.1016/j.aspen.2018.03.004

[B109] Uliana M Bonato L Minelli A (2007) The Mecistocephalidae of the Japanese and Taiwanese islands (Chilopoda: Geophilomorpha). Zootaxa 1396(1): 1–84. 10.11646/zootaxa.1396.1.1

[B110] Verhoeff KW (1928) Geophilomorphen-Beiträge und eine *Lithobius*-Form. Mitteilungen aus dem Zoologischen Museum in Berlin 14: 229–286. 10.1002/mmnz.4830140202

[B111] Verhoeff KW (1934) Beiträge zur Systematik und Geographie der Chilopoden. Zoologische Jahrbücher. Abteilung für Systematik 66: 1–112.

[B112] Verhoeff KW (1935) Über *Scolioplanes* (Chilopoda). Zoologischer Anzeiger 111: 10–23.

[B113] Volkova YuS (2016) An annotated catalogue of geophilomorph centipedes (Chilopoda, Geophilomorpha) from the European part of Russia. Zoologicheskii Zhurnal 95(6): 669–678. [In Russian, with English summary] 10.1134/S0013873816040138

[B114] Vorobiova IG (1999) Ecological and faunistic characteristics of myriapod populations in the mid-flow region of Yenissei River. In: Striganova BR (Ed.) Problemy pochvennoi zoologii.Materialy II (XII) Vserossiiskogo soveshchaniya po pochvennoi zoologii. KMK Press, Moscow, 33–34. [In Russian]

[B115] Vorobiova IG Rybalov LB Rossolimo TE Zalesskaja NT (2002) Zonal and landscape distribution of the myriapod fauna and populations (Myriapoda) in the Yenisei River basin. In: Izuchenie, sokhranenie i vosstanovlenie bioraznoobraziya ekosistem na Yeniseiskom ekologicheskom transekte: Zhivotnyi mir, etno-ekologicheskie issledovniya, 2. IEE RAS Publisher, Moscow, 60–71. [In Russian]

[B116] Zalesskaja NT Titova LP Golovatch SI (1982) The myriapod fauna of the Moscow Region. In: Ghilarov MS (Ed.) Pochvennye bespozvonochnye Moskovskoi oblasti.Nauka Publisher, Moscow, 179–200. [In Russian]

[B117] Zuev RV (2016) Centipedes (Chilopoda) from the Stavropol Territory, northern Caucasus, Russia. Arthropoda Selecta 25(1): 23–38. 10.15298/arthsel.25.1.03

[B118] Zuev RV Evsyukov AP (2016) Centipedes (Chilopoda) from the Rostov-on-Don Region, southern Russia. Russian Entomological Journal 25(4): 417–426. 10.15298/rusentj.25.4.12

